# Radiomics and Artificial Intelligence in Ovarian Endometriosis Imaging: A Systematic Review and Critical Appraisal of Emerging Evidence

**DOI:** 10.3390/bioengineering13080855

**Published:** 2026-07-24

**Authors:** Teodora Telecan, Roxana-Denisa Capraș, Simina Teodora Poenar, Roxana Sevan-Libotean, Adriana Ioana Gaia-Oltean, Carmen-Bianca Crivii, Alexandru-Florin Badea

**Affiliations:** 1 Department of Anatomy and Embryology, “Iuliu Hațieganu” University of Medicine and Pharmacy, 400012 Cluj-Napoca, Romania; t.telecan@gmail.com (T.T.); poenar_simina_teodora@elearn.umfcluj.ro (S.T.P.); sevanliboteanroxana@elearn.umfcluj.ro (R.S.-L.); bianca.crivii@umfcluj.ro (C.-B.C.); afbadea84@gmail.com (A.-F.B.); 2 Department of Pathology, County Emergency Clinical Hospital, 400347 Cluj-Napoca, Romania; 3 Department of Obstetrics and Gynecology, “Iuliu Hațieganu” University of Medicine and Pharmacy, 400347 Cluj-Napoca, Romania; oltean_adriana_ioana@yahoo.com; 4 Department of Obstetrics and Gynecology, “Regina Maria” Hospital, 400609 Cluj-Napoca, Romania

**Keywords:** ovarian endometriosis, endometrioma, radiomics, texture analysis, ultrasound, magnetic resonance imaging, computed tomography, artificial intelligence

## Abstract

Background: Ovarian endometriosis is a common manifestation of endometriosis associated with chronic pelvic pain and infertility. Although transvaginal ultrasound and magnetic resonance imaging are central to diagnosis, challenging cases may benefit from more objective imaging biomarkers. Radiomics and artificial intelligence (AI) have emerged as promising approaches for quantitative lesion characterization. Objective: To systematically review current evidence on radiomics and AI for imaging-based evaluation of ovarian endometriosis. Methods: PubMed, Scopus, and Web of Science were systematically searched from inception to 31 March 2026. Studies investigating radiomics or AI applied to ultrasound, computed tomography, or magnetic resonance imaging for ovarian endometriosis were included. Study characteristics, radiomics methodologies, and diagnostic performance were qualitatively synthesized. Results: Nine studies met the inclusion criteria, including five ultrasound-, two computed tomography-, and two magnetic resonance imaging-based investigations. Among the seven studies addressing diagnostic classification, five reported diagnostic accuracy directly, with values ranging from 83.7% to 96.8%. The remaining two studies addressed automated lesion segmentation rather than diagnostic classification. Integrated radiomics nomograms improved diagnostic performance compared with clinical assessment alone and with less experienced readers. However, the evidence base was limited by small, predominantly retrospective, single-center studies with substantial methodological heterogeneity. Conclusions: Radiomics shows promise as a complementary imaging tool for ovarian endometriosis, but current evidence remains preliminary. Prospective multicenter validation, methodological standardization, and transparent reporting are required before routine clinical implementation.

## 1. Introduction

Endometriosis is a chronic, hormonal-dependent inflammatory condition defined by the presence of endometrial-like tissue outside the uterine cavity, affecting more than 190 million women of reproductive age worldwide [1]. Ovarian endometriosis, characterized by the formation of endometriotic cysts (endometriomas) within the ovary, represents one of the most common subphenotypes of the disease, occurring in approximately 24% of women with endometriosis as reported in large population-based cohorts [2]. The clinical relevance of ovarian endometriosis is substantial, given its strong association with chronic pelvic pain, progressive dysmenorrhea, and infertility, driven by both mechanical distortion and local inflammatory damage to ovarian tissue, which may impair ovarian reserve independent of cyst size [3,4]. In addition, ovarian endometriomas are associated with an increased risk of epithelial ovarian cancer, particularly clear cell and endometrioid histotypes [5], with large population-based studies reporting adjusted hazard ratios for type I ovarian cancer of up to 19-fold compared with women without endometriosis [6].

From a diagnostic perspective, the imaging diagnosis of typical ovarian endometriomas is generally straightforward in experienced hands; however, clinically relevant challenges arise when imaging findings are atypical or when complex adnexal masses are encountered. Endometriomas may mimic a variety of benign functional cysts, particularly hemorrhagic corpus luteum cysts, as well as other ovarian pathologies such as dermoid cysts, tubo-ovarian inflammatory masses, or, less frequently, cystic ovarian malignancies [7]. Transvaginal ultrasound and magnetic resonance imaging demonstrate high diagnostic performance for typical ovarian endometriomas, with reported sensitivities and specificities frequently exceeding 85–90% in expert settings [8]. Nevertheless, diagnostic confidence decreases in cases with atypical imaging features, complex adnexal morphology, or coexisting pelvic pathology, where overlapping appearances may lead to uncertainty and variable interobserver agreement. In such scenarios, imaging findings play a central role in guiding clinical decision-making, particularly when balancing conservative management against surgical intervention [9]. Misclassification may have important clinical consequences, including unnecessary surgical intervention with potential loss of ovarian reserve, delayed fertility management, or, conversely, failure to promptly recognize malignant disease [10]. Consequently, improving the noninvasive characterization of cystic ovarian lesions represents a clinically meaningful objective in the management of ovarian endometriosis.

The imaging diagnosis of ovarian endometriosis is primarily based on transvaginal ultrasound, which is recommended as the first-line modality by major international guidelines, whereas magnetic resonance imaging serves as a reliable second-line tool for equivocal cases and preoperative assessment, with endometriomas classically demonstrating T1 hyperintensity and T2 hypointensity (“shading”). Beyond its diagnostic role, T2-weighted imaging is also of particular technical relevance for radiomics workflows: T2-weighted sequences generally provide higher soft-tissue contrast than T1-weighted sequences, which has been shown to improve automated and semi-automated segmentation performance in other anatomical contexts, and may similarly facilitate more reliable lesion delineation and feature extraction in ovarian endometriomas [11,12,13]. Computed tomography is not routinely used due to limited sensitivity but may be helpful in selected acute or complex scenarios, such as suspected cyst rupture or when mapping deep infiltrative disease [14,15].

Despite their central role in clinical practice, conventional imaging techniques for ovarian endometriosis have important limitations. Transvaginal ultrasound, whilst widely available and cost-effective, is highly operator-dependent, with diagnostic accuracy influenced by sonographer expertise, equipment quality, and patient-related factors such as body habitus and complex pelvic anatomy [7]. Magnetic resonance imaging offers improved tissue characterization, comprehensive disease mapping malignant potential assessment; however, its use is constrained by higher cost and limited accessibility, and its performance depends on dedicated endometriosis protocols and experienced interpretation [16]. Although histopathological confirmation remains the reference standard in many research settings, contemporary clinical management increasingly relies on expert imaging and clinical findings, particularly when surgery is not immediately indicated. Consequently, the principal limitation of imaging lies not in the absence of histological confirmation itself, but in the diagnostic uncertainty that persists in selected equivocal or complex cases. Furthermore, overlap in imaging features between ovarian endometriomas and other adnexal masses—such as hemorrhagic cysts or benign ovarian lesions—may lead to diagnostic uncertainty in selected patients [1].

In light of the recognized limitations of conventional imaging, radiomics has emerged as a promising quantitative approach capable of extending the diagnostic potential of routinely acquired imaging examinations in ovarian endometriosis. Radiomics involves the computational extraction of numerous quantitative features from medical images, capturing lesion shape, signal distribution, and internal heterogeneity beyond what can be assessed by visual interpretation alone [17]. In parallel, artificial intelligence (AI)—a broad computational framework encompassing algorithms capable of learning patterns from data to support classification, prediction, or decision-making tasks—has become increasingly integrated with radiomics workflows [18,19]. Within AI, deep learning (DL) architectures, most notably convolutional neural networks, enable automated feature learning directly from raw imaging data, in contrast to conventional radiomics pipelines that rely on predefined, handcrafted features requiring explicit segmentation and extraction steps [20,21]. This segmentation step typically relies on manual or semi-automatic lesion delineation, which demands substantial domain expertise and is time-intensive, particularly for small or low-contrast structures—burdens that have been documented in the broader neuroimaging literature, where manual segmentation of comparably challenging deep-brain structures has been reported to require dedicated specialist time for every new case and to yield only moderate interrater agreement even between trained experts (e.g., Dice = 0.63 ± 0.10) [22,23]. These constraints represent a practical limit on the scalability and reproducibility of conventional radiomics workflows, particularly for MRI. Convolutional operations underlie both paradigms, though in distinct roles. In conventional radiomics pipelines, standardized convolutional filters—such as Laplacian-of-Gaussian and wavelet transforms—are applied as fixed, non-learned image transformations to enhance specific textural or edge characteristics prior to handcrafted feature extraction [18,20]. In deep learning architectures, by contrast, convolutional layers consist of learnable filter kernels whose weights are optimized during training, enabling the network to automatically extract hierarchical spatial features directly from the raw image without a separate, explicitly defined filtering step.

Although the concept was formally introduced in 2012 [24], its application to ovarian endometriomas is relatively recent, with the first radiomics-based studies published in 2020 [25]. Since then, radiomic analyses have been explored across multiple imaging modalities—most notably ultrasound and MRI—with the aim of improving lesion characterization, supporting differential diagnosis, and evaluating potential indicators of malignant transformation. Ultrasound-based radiomics presents particular technical challenges, as speckle noise, low soft-tissue contrast, and acoustic shadowing can distort lesion boundaries and destabilize the texture matrices from which radiomic features are computed, making artifact-aware preprocessing a prerequisite rather than an optional step in ultrasound radiomics pipelines [26]. In clinical research, radiomic features are frequently incorporated into multivariable prediction models, often presented as nomograms—graphical tools that combine imaging, clinical, and laboratory variables to estimate the probability of a specific diagnosis or outcome for an individual patient [27]. As a noninvasive preoperative analytical tool derived from routinely acquired imaging data, radiomics has the potential to contribute to imaging-based risk stratification and individualized clinical decision-making, particularly in patients with equivocal imaging findings [28].

This premise, however, presupposes the availability of accurate and reproducible lesion contours, which is not a trivial assumption in practice. Manual segmentation is subject to inter- and intra-rater variability, particularly for lesions with atypical or ambiguous margins [26]. Automated segmentation, whilst more scalable, may be especially prone to boundary errors in precisely the equivocal or atypical cases in which radiomics is expected to add the greatest clinical value; because radiomic features are computed directly from the segmented region, an inaccurate boundary propagates mathematically into every downstream feature, undermining the reliability of any resulting clinical decision-support tool. However, the current evidence remains limited and methodologically heterogeneous, with variability in image acquisition, segmentation strategies, feature extraction pipelines, validation approaches, and reporting standards limiting reproducibility and direct comparison across studies. Consequently, the extent to which radiomics can be translated into routine clinical practice remains uncertain, underscoring the need for a critical evaluation of the currently available evidence.

Ovarian endometriosis has previously been the subject of several narrative reviews, addressing pathogenesis and malignant transformation risk, histopathological features, and surgical or medical management strategies. However, none of these prior works has systematically synthesized evidence on the use of radiomics or artificial intelligence for imaging-based characterization of ovarian endometriosis. Table 1 summarizes the scope of these related works relative to the present review, highlighting the specific gap addressed here: a systematic, PRISMA-guided synthesis of radiomics- and AI-based diagnostic approaches across ultrasound, computed tomography, and magnetic resonance imaging.

The objective of this review is therefore to critically evaluate the current evidence regarding radiomics and artificial intelligence applied to ovarian endometriosis imaging, with particular emphasis on diagnostic performance, methodological quality, and potential clinical applicability. Specifically, this review addressed the following research questions:

1. What is the diagnostic performance of radiomics- and AI-based models for characterizing ovarian endometriosis across ultrasound, computed tomography, and magnetic resonance imaging?
2. What methodological approaches—including segmentation, feature extraction, and model validation strategies—have been employed across studies, and to what extent does methodological heterogeneity limit comparability?
3. What are the principal methodological and translational barriers currently limiting the clinical implementation of these approaches?

**Figure 1:**
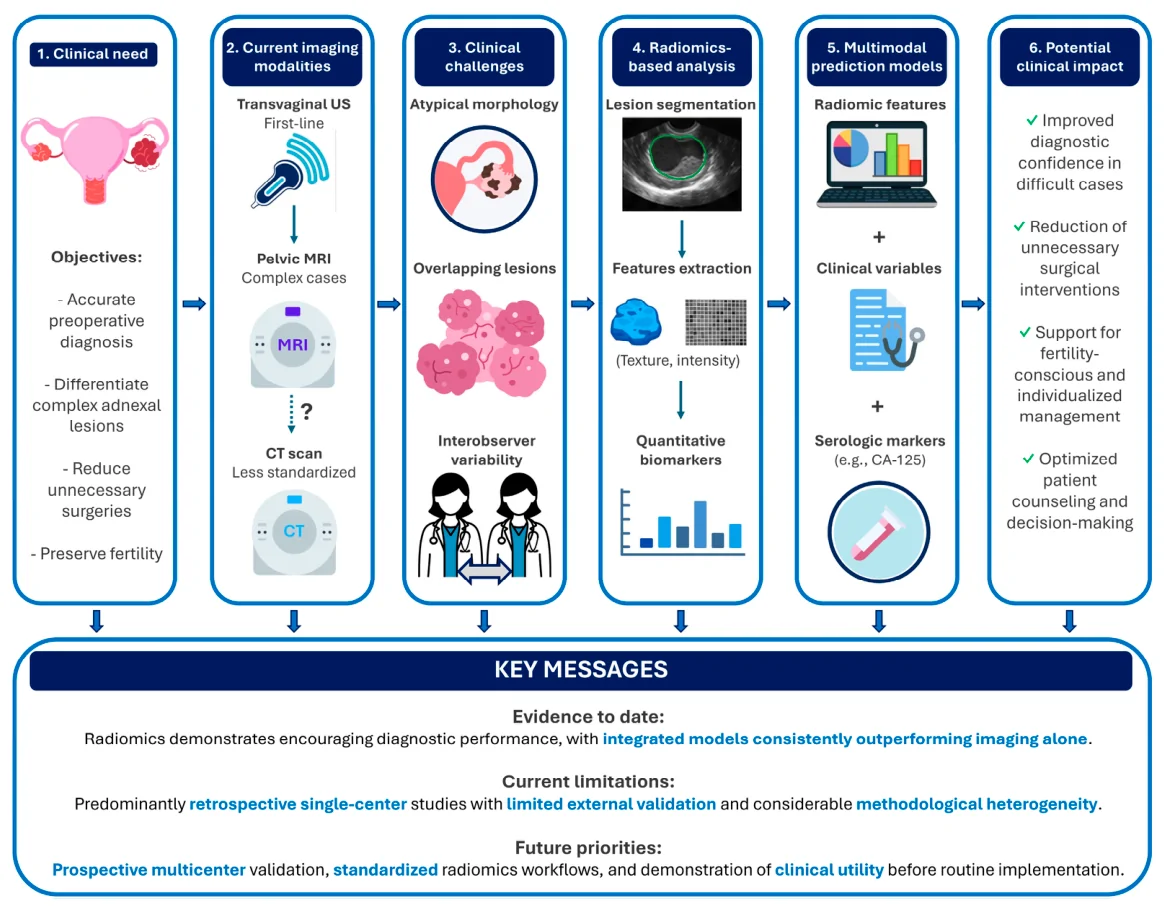
Proposed framework for the clinical integration of radiomics in ovarian endometriosis, outlining clinical objectives, current imaging modalities, associated diagnostic challenges, the radiomics-based analysis pipeline, multimodal prediction models integrating radiomic, clinical, and serologic data, and potential clinical impact.

Beyond summarizing the available literature, this review aims to provide a structured synthesis of methodological approaches, imaging modality-specific applications, and validation strategies in order to clarify the current level of clinical maturity of radiomics in this field. Furthermore, it seeks to identify the principal methodological and translational challenges that must be addressed before these approaches can be considered for routine clinical implementation and to outline realistic directions for future research. A conceptual overview of the proposed clinical integration of radiomics in ovarian endometriosis is presented in Figure 1.

The remainder of this article is organized as follows: Section 2 describes the search strategy, eligibility criteria, and methodological quality assessment employed in this systematic review. Section 3 presents the results of study selection and synthesizes findings by imaging modality, covering ultrasound-, computed tomography-, and MRI-based radiomics. Section 4 discusses the clinical need for improved preoperative characterization, the principal findings and methodological maturity of the current evidence, and its readiness for clinical translation. Section 5 outlines the limitations of the current evidence base, and Section 6 presents concluding remarks and directions for future research.

## 2. Materials and Methods

This systematic review was conducted in accordance with the PRISMA 2020 statement [35]. The review protocol was retrospectively registered on the Open Science Framework (OSF; Registration: https://osf.io/hryvc/, accessed on 26 June 2026) to improve methodological transparency and provide a permanent public record of the review methodology. A comprehensive literature search was independently performed by two reviewers (T.T. and S.T.P.) using PubMed, Scopus, and Web of Science (WoS) to identify original studies investigating radiomics, texture analysis, or artificial intelligence-based imaging approaches applied to ovarian endometriosis across different imaging modalities. The screening process was carried out independently by the same reviewers, beginning with titles and abstracts and followed by full-text eligibility assessment. Any disagreements regarding study inclusion were resolved through discussion and consensus, with consultation of a third, senior reviewer (A.-F.B.) when required. Reviewers were not blinded to authorship, institutional affiliations, or journal information, as such details are inherent to published articles; however, standardized eligibility criteria and predefined data extraction forms were employed to limit subjective bias. Search across all databases was conducted in parallel and completed within the same search timeframe, from inception to 31 March 2026. Only studies published in English with full-text availability were considered eligible for inclusion.

A comprehensive literature search was conducted in PubMed, Scopus, and Web of Science using database-specific controlled vocabulary and free-text terms related to ovarian endometriosis and radiomics-based imaging analysis. The complete electronic search strategies, including database-specific search fields, Boolean syntax, search limits, search dates, and the number of records retrieved, are provided in Table S1.

Database searches were initially conducted in September 2025. No publication date limits were applied during this initial search. A temporal restriction to studies published within the last 10 years was subsequently applied during the title/abstract screening and full-text eligibility assessment stages, in accordance with the PRISMA flow diagram, to ensure relevance to contemporary imaging technologies and current clinical practice. This 10-year timeframe was selected to capture studies employing modern radiomics pipelines and feature definitions aligned with current methodological standards, including Image Biomarker Standardization Initiative (IBSI)-compliant workflows [36]. Earlier texture-analysis studies predating standardized radiomics frameworks were therefore considered outside the scope of the present review. To confirm the currency of the evidence base prior to submission, the search was updated in March 2026 using identical search strings and databases; no additional eligible studies were identified. To maintain a single, reproducible eligibility window aligned with the final search date, studies published before 2016 were excluded, consistent with the exclusion criteria reported in the exclusion criteria above. The same temporal criterion was applied consistently across all imaging modalities and analytical objectives. To further enhance search completeness, the reference lists of all included studies were manually reviewed. Any disagreements between reviewers were resolved through discussion and consensus, with arbitration by the senior author when necessary.

Studies were considered eligible if they investigated the performance of radiomic features or artificial intelligence-based decision support systems in the characterization of ovarian endometriomas (synonymously, endometriotic cysts). Eligible studies were required to establish the diagnosis of ovarian endometrioma through histopathological confirmation obtained after surgical excision, or through an equivalent, adequately documented reference standard such as expert clinical and sonographic/radiological consensus with imaging follow-up. This reflects accepted diagnostic practice in this literature, where surgical excision is not performed for every lesion and where prior work has shown that restricting inclusion to histologically confirmed cases alone can introduce greater selection bias [25]. The specific reference standard used in each included study is reported in the corresponding modality-specific results tables. Radiomics-based analyses aimed at differentiating endometriomas from other ovarian cystic lesions or at providing lesion characterization were considered within scope.

Studies were excluded if they met any of the following criteria:

- Full text not available in English;
- Designed as a systematic review, meta-analysis, narrative review, comment, letter to the editor, or conference/meeting abstract;
- Published before 2016;
- Conducted on animal models or experimental (non-clinical) settings;
- Did not describe radiomics-based, texture analysis, or artificial intelligence methodologies;
- Did not report separately extractable radiomics, texture-analysis, or artificial-intelligence results for ovarian endometriomas.

Data extraction was carried out independently by two reviewers using a predefined and standardized extraction template. The collected data encompassed general study characteristics (year of publication, study design, sample size, and patient population), imaging modality and acquisition protocols, lesion segmentation methods, and details of the radiomics pipeline.

Additional variables included the incorporation of clinical or biological parameters, the reference standard used for outcome definition, validation strategies, and reported performance metrics, such as accuracy, sensitivity, specificity, and area under the receiver operating characteristic curve (AUC), when available. When available, additional methodological variables—including feature normalization or standardization procedures, segmentation reproducibility, model calibration, decision-curve analysis, and reporting of methodological standards—were also extracted. Any disagreements between reviewers during data extraction were resolved through discussion and consensus, with involvement of the senior reviewer when required. Owing to the methodological and clinical heterogeneity of the included studies, findings were synthesized using a qualitative approach.

A formal quantitative meta-analysis was not undertaken because of substantial methodological and clinical heterogeneity across the included studies. Important differences were observed in imaging modality, study objectives, segmentation methodology, radiomics pipelines, machine learning algorithms, validation strategies, and reported outcome measures. Furthermore, the limited number of studies within each imaging modality and the frequent absence of external validation precluded statistically meaningful quantitative synthesis. Accordingly, a qualitative synthesis was considered the most appropriate approach.

**Figure 2:**
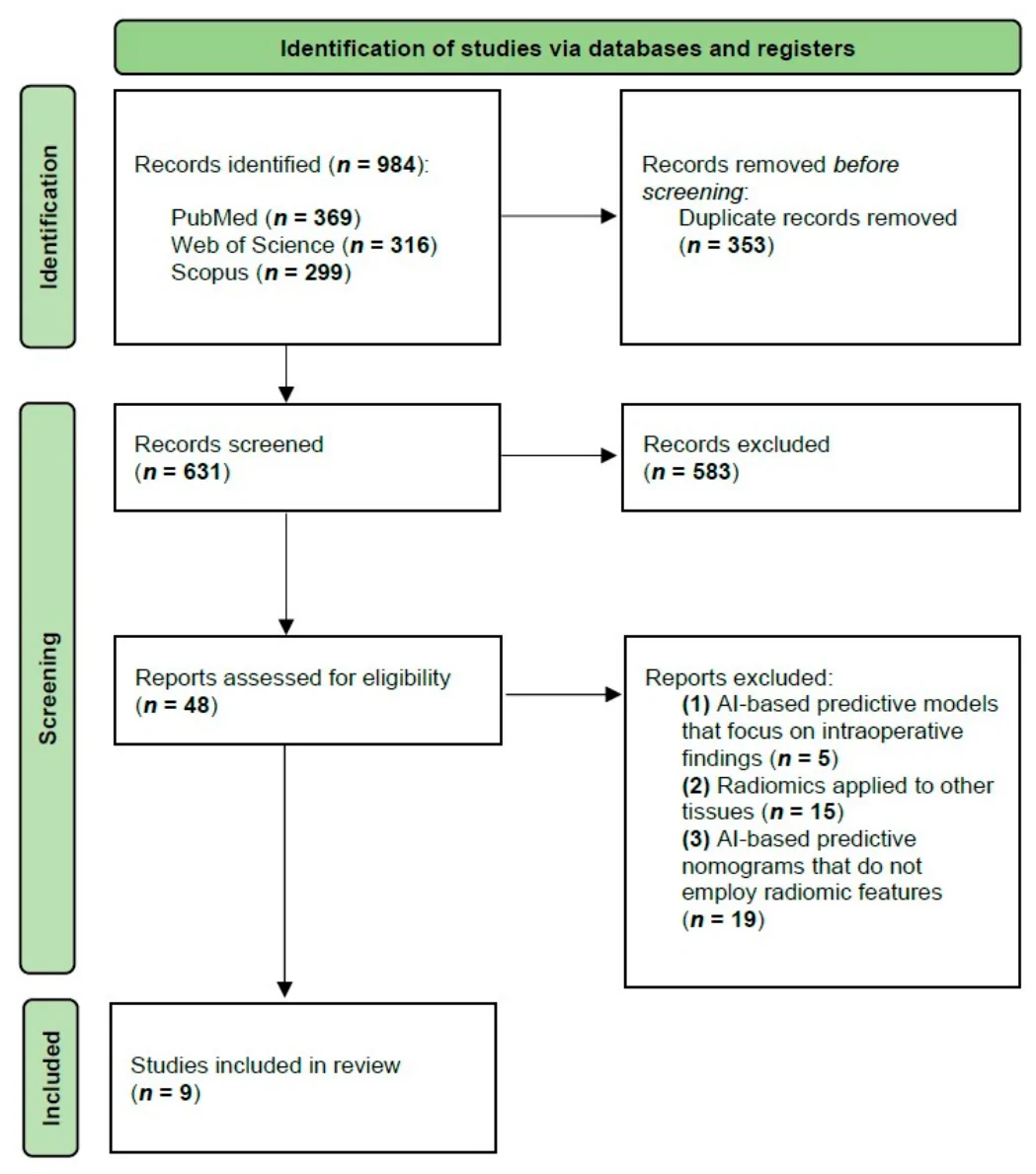
PRISMA flow diagram of the study selection process.

The study selection process is summarized using a Preferred Reporting Items for Systematic Reviews and Meta-Analyses (PRISMA) flow diagram (Figure 2). In addition, the completed PRISMA 2020 checklist is provided in Table S2 [35].

The methodological quality and risk of bias of the included studies were evaluated using the Quality Assessment of Diagnostic Accuracy Studies-2 (QUADAS-2) tool [37]. In this review, the QUADAS-2 framework was applied independently by two reviewers to all eligible studies using predefined signaling questions. Any discrepancies in judgments were resolved through discussion and consensus, with consultation of the senior author when required. Risk-of-bias assessment was performed to facilitate interpretation of the overall quality of the evidence rather than to determine study eligibility.

Methodological characteristics specific to radiomics studies—including segmentation reproducibility, feature standardization, validation strategy, model calibration, decision-curve analysis and adherence to reporting frameworks such as TRIPOD+AI [38]—were extracted descriptively to facilitate critical appraisal of methodological quality beyond conventional diagnostic accuracy assessment.

## 3. Results

### 3.1. Study Selection and Characteristics

The database search identified a total of 984 records, including 369 from PubMed, 316 from Web of Science, and 299 from Scopus. After removal of 353 duplicate records, 631 unique records remained and were screened based on titles and abstracts. Of these, 583 records were excluded for not meeting the inclusion criteria. The full texts of 48 articles were subsequently assessed for eligibility. Following full-text review, 39 studies were excluded for the following reasons: use of artificial intelligence-based predictive models focusing on intraoperative findings without imaging radiomics features (*n* = 5), application of radiomics to tissues other than ovarian endometriotic lesions (*n* = 15), or development of predictive nomograms that did not incorporate radiomic features (*n* = 19).

In total, 9 studies were retained and formed the basis of qualitative synthesis. With respect to imaging modalities, five studies were based on ultrasound imaging, two employed computed tomography and two utilized magnetic resonance imaging. The database search was originally conducted in September 2025 and updated in March 2026 using identical search strategies across all three databases; no additional eligible studies published in the interim met the predefined inclusion criteria.

**Figure 3:**
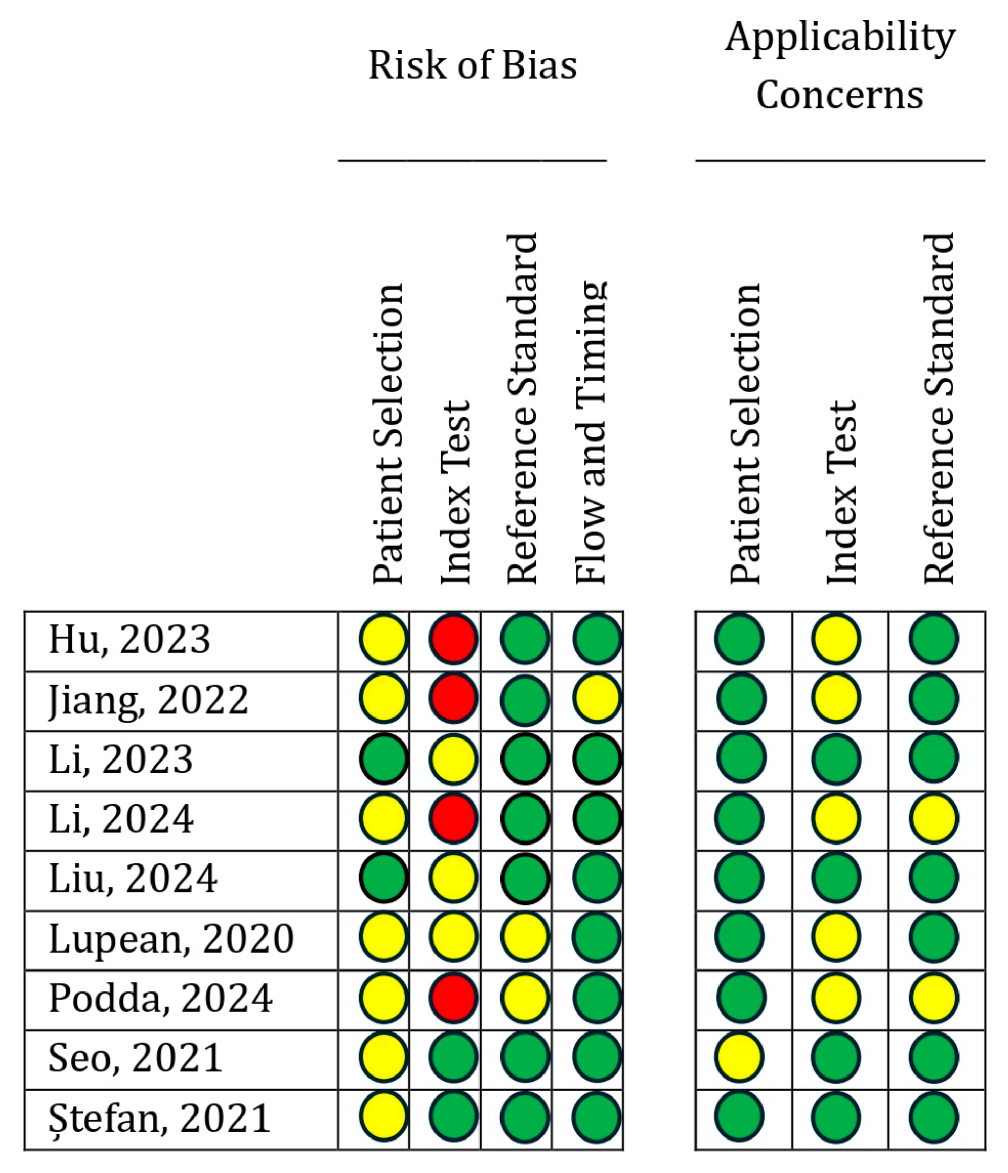
Detailed QUADAS-2 evaluation of methodological quality for each included study assessing the diagnostic accuracy of radiomics models [25,39,40,41,42,43,44,45,46]. Green = low risk of bias; Yellow = unclear risk of bias; Red = high risk of bias.

**Figure 4:**
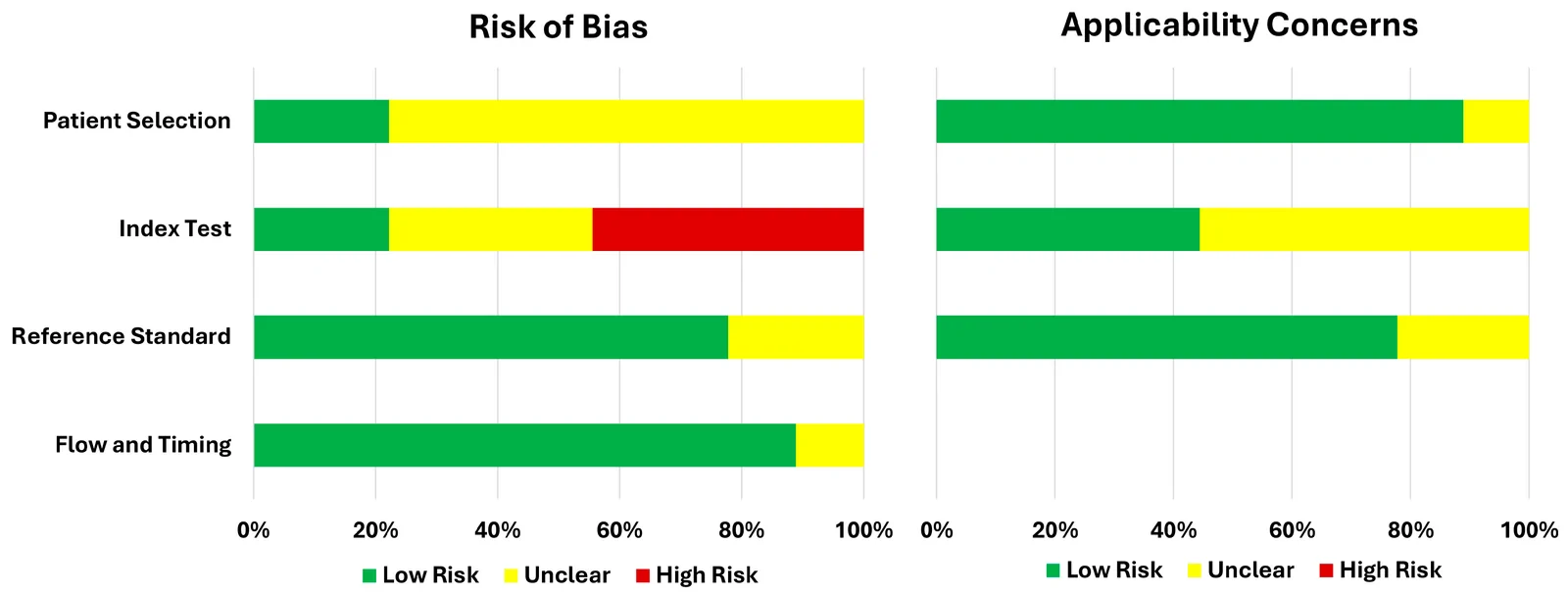
Summary of QUADAS-2 risk of bias and applicability concerns across all included studies, presented as proportions for each domain.

The QUADAS-2 risk of bias assessment is summarized in Figure 3 (per-study evaluation) and Figure 4 (domain-level overview). Overall, the included studies demonstrated a low risk of bias in the reference standard and flow and timing domains, reflecting consistent use of appropriate reference standards and study conduct. In contrast, the index test domain showed a higher proportion of studies at high or unclear risk of bias, primarily due to internal model development, primarily due to reliance on internally developed models, limited external validation, and incomplete reporting of blinding procedures. Patient selection was frequently rated as unclear owing to retrospective single-center designs and insufficient reporting of enrollment strategies. Applicability concerns were generally low across all domains, indicating good alignment between study populations, index tests, and reference standards and the clinical context of ovarian endometriosis. Detailed domain-level judgments and the rationale for each QUADAS-2 assessment are provided in Table S3. In addition to this conventional risk-of-bias assessment, a radiomics-specific methodological quality appraisal of the included studies is presented in Section 3.5.

### 3.2. Ultrasound-Based Radiomics

A total of five studies met the inclusion criteria and investigated the diagnostic performance of ultrasound-based radiomics and artificial intelligence approaches for the characterization of ovarian endometriotic cysts. The main characteristics and key findings of these studies are summarized in Table 2. All included investigations were retrospective, single-center studies, with sample sizes ranging from 56 to 407 cases. Across studies, ovarian endometriomas were differentiated from a spectrum of benign adnexal conditions, including hemorrhagic ovarian cysts, tubo-ovarian abscesses, and dermoid cysts. All studies employed transvaginal ultrasound as the primary imaging modality, with two studies additionally incorporating transabdominal ultrasound and one study utilizing a transrectal approach. Lesion segmentation strategies varied, comprising manual segmentation in three studies, semi-automatic segmentation in one study, and fully automatic segmentation in one study. The reference standard varied across studies, comprising histopathological confirmation in two studies and a mixed or expert clinical/sonographic reference standard in the other two (see Table 2). Among the four studies addressing diagnostic classification, reported performance was consistently high: area under the curve (AUC) was reported in three of the four studies, ranging from 0.967 to 1.00 (not reported for Li et al. [41]); sensitivity ranged from 87.9% to 100%; specificity ranged from 87.15% to 100%; and accuracy ranged from 91.36% to 96.8%, although performance estimates were generally derived from internal validation only. The remaining study addressed automated lesion segmentation rather than diagnostic classification and is summarized separately below.

Two ultrasound-based studies focused specifically on the automated segmentation of ovarian endometriotic lesions. Podda et al. [43] developed a deep learning ensemble model designed to improve lesion delineation on transvaginal ultrasound images using expert-annotated pixel-level segmentations. The approach combined multiple convolutional neural networks trained at different spatial resolutions, allowing the model to capture both global cyst morphology and local boundary characteristics. Model performance was evaluated using five-fold cross-validation and quantified by standard segmentation metrics, including the Dice coefficient, Jaccard index, and detection accuracy. The best-performing ensemble achieved a Dice coefficient of 82% and a Jaccard index of 71%, outperforming the individual single-resolution models. The authors reported that the ensemble model achieved higher segmentation performance than the individual single-scale models, suggesting potential utility for automated lesion delineation within ultrasound-based radiomics workflows.

In contrast, Li et al. [41] developed a large-scale deep learning framework designed to perform both lesion segmentation and binary classification of ovarian endometriomas versus other cystic adnexal lesions on ultrasound. This study included 406 patients. The study included 1601 images: 1501 were used for model development and cross-validation, while a separate set of 100 images was used for hold-out testing, representing the largest dataset among the included ultrasound investigations. Segmentation was based on an advanced neural network architecture capable of capturing complex spatial relationships within ultrasound images, with segmentation outputs subsequently integrated into the classification process to enhance lesion-focused prediction. Ground truth annotations were provided by experienced sonographers. Model development involved five-fold cross-validation and additional evaluation on an independent hold-out test set. For segmentation, the model achieved a Dice coefficient of 85.42%, outperforming comparator approaches. The reported results indicate that deep learning-based segmentation and classification can achieve high performance when trained on relatively large, well-annotated datasets.

One study specifically explored the role of ultrasound-based radiomic texture analysis in differentiating ovarian endometriomas from hemorrhagic ovarian cysts. Ștefan et al. [39] conducted a retrospective analysis of transvaginal ultrasound examinations acquired using a standardized protocol on a single imaging system. A total of 275 handcrafted texture features were extracted from semi-automatically segmented two-dimensional regions of interest following image preprocessing and feature selection procedures. After reproducibility filtering, seven texture parameters were retained and incorporated into a multivariable regression model. The resulting radiomics model demonstrated perfect apparent diagnostic performance, achieving an AUC of 1.00 with 100% sensitivity and specificity, substantially outperforming conventional grayscale ultrasound features reported by the authors. Whereas these findings highlight the potential discriminative value of quantitative texture analysis, the exceptionally high performance should be interpreted cautiously in light of the small sample size, single-center design, lack of external validation, and the use of feature selection and model evaluation within the same dataset, all of which may contribute to optimistic estimates of diagnostic accuracy.

Additionally, two studies explored ultrasound-based artificial intelligence approaches that combined imaging-derived information with clinical or biochemical parameters to develop integrated diagnostic frameworks. Liu et al. [42] proposed a radiomics-based nomogram aimed at differentiating ovarian endometriomas from dermoid cysts by incorporating quantitative ultrasound features together with selected clinical variables. Radiomic features were extracted from manually segmented transvaginal ultrasound images and combined with parameters such as patient age and lesion size within a multivariable prediction model. The integrated nomogram demonstrated superior diagnostic performance compared with either the radiomics signature or clinical assessment alone, achieving an AUC of 0.987, sensitivity of 88.0%, specificity of 100%, and accuracy of 95.1% in the test cohort, compared with an AUC of 0.967, sensitivity of 87.9%, specificity of 97.1%, and accuracy of 92.6% for the radiomics signature alone. Within this study, integration of quantitative imaging features with clinical variables was associated with higher diagnostic performance than either component alone.

Lastly, Hu et al. [40] investigated a deep learning-based classification approach for differentiating ovarian endometriomas from tubo-ovarian abscesses. Model performance was evaluated in comparison with experienced sonographers and with the biochemical marker CA-125, rather than through formal integration of clinical variables into a combined prediction model. The study used both transvaginal and transabdominal ultrasound images, and several neural network architectures were trained and tested on an independent cohort with histopathological confirmation. The best-performing model achieved an AUC of 0.986, with overall accuracy of 96.8%, sensitivity of 90%, and specificity of 100%, significantly outperforming ultrasound physicians (AUC range 0.683–0.781) and CA-125 alone (AUC 0.564). Although clinical and laboratory variables were not incorporated directly into the prediction model, comparison with these approaches highlighted the high reported discriminative performance of the image-based deep learning model within this dataset.

Collectively, the ultrasound-based studies investigated automated segmentation, radiomics-based texture analysis, deep learning classification, and integrated prediction models combining imaging with clinical variables. Among the four diagnostic classification studies, reported performance was generally high, although study designs, patient populations, imaging protocols, and validation strategies varied considerably. The segmentation study [43] addressed a distinct methodological objective—automated lesion delineation—and its detection accuracy is not directly comparable to the diagnostic performance metrics reported above. Most investigations were retrospective, single-center studies relying primarily on internal validation, with only limited assessment of model calibration or external validation. These findings illustrate the broad range of ultrasound-based artificial intelligence applications currently being explored for ovarian endometriosis.

### 3.3. Computed Tomography-Based Radiomics

A total of two studies met the inclusion criteria and evaluated the diagnostic performance of CT-based radiomics and artificial intelligence approaches for the characterization of ovarian endometriotic cysts. The main characteristics and key findings of these studies are summarized in Table 3. Both investigations were retrospective, single-center studies, with sample sizes of 135 and 287 patients, respectively. In these studies, ovarian endometriomas were differentiated from other benign adnexal lesions, including serous and mucinous cystadenomas as well as follicular cysts. In both cases, imaging was performed using contrast-enhanced computed tomography acquired in the portal venous phase, and lesion delineation was achieved through manual segmentation of the cystic regions. The reference standard was uniformly based on histopathological confirmation following surgical removal of the cyst or ovary. Reported diagnostic performance across studies was moderate to high, with sensitivity ranging from 72.7% to 90.0%, specificity from 87.7% to 89.0%, and accuracy from 83.7% to 88.5%, reflecting the potential utility of CT-based quantitative analysis for the evaluation of ovarian endometriotic cysts.

Seo et al. [44] conducted a retrospective single-center study assessing the added value of CT-based texture analysis compared with conventional contrast-enhanced CT for differentiating benign ovarian cystic lesions, including endometriotic cysts. The study included 135 surgically confirmed lesions evaluated by two readers with different levels of experience. Conventional CT assessment relied on qualitative and quantitative features such as cyst wall characteristics, internal attenuation, and lesion morphology, whereas texture analysis was performed using manually defined regions of interest and extraction of first-order statistical parameters. Diagnostic models combining conventional imaging features with texture-derived metrics demonstrated only modest improvements for the experienced radiologist but resulted in a more notable increase in sensitivity and overall diagnostic accuracy for the less experienced reader. Within this study, the addition of quantitative CT texture features was associated with improved diagnostic performance, particularly for the less experienced reader, suggesting that texture analysis may provide complementary information in selected diagnostic scenarios. Although the primary aim of this study was to improve differentiation of mucinous cystadenoma, its design included four well-defined, histopathologically confirmed diagnostic categories—including 43 surgically excised endometriotic cysts—with dedicated CT texture-analysis data reported and compared across all categories. This provides direct, extractable evidence on the radiomic characterization of ovarian endometriotic cysts against other benign adnexal lesions, meeting this review’s inclusion criteria on data-relevance grounds independent of the source study’s stated primary objective.

Subsequently, Li et al. [45] further explored the role of CT radiomics by implementing a volumetric analysis strategy for the preoperative differentiation of ovarian endometriotic cysts from cystadenomas, with a focus on developing an integrated radiomics–clinical prediction model. Quantitative features were extracted from manually segmented three-dimensional lesion volumes on contrast-enhanced CT, enabling assessment of lesion morphology, attenuation patterns, and internal heterogeneity. Feature robustness was ensured through inter- and intraobserver agreement testing, followed by statistical selection procedures that yielded a compact radiomics signature. This imaging-derived signature was further combined with serum CA-125 levels to construct a multivariable nomogram estimating the probability of an ovarian endometriotic cyst. In the validation cohort, the combined model demonstrated superior performance compared with radiomics alone, clinical assessment alone, and radiologist interpretation, achieving an AUC of 0.942, sensitivity of 90.0%, specificity of 87.7%, and overall accuracy of 88.5%. Within the validation cohort, integration of CT-derived radiomics features with serum CA-125 was associated with higher diagnostic performance than radiomics, clinical assessment, or radiologist interpretation alone.

The available CT studies primarily investigated quantitative image analysis for the preoperative differentiation of ovarian endometriotic cysts from other cystic adnexal lesions, using either conventional texture analysis or volumetric radiomics approaches. Although the reported diagnostic performance was generally high, both studies were retrospective single-center investigations that differed substantially in imaging protocols, segmentation methodology, predictive modelling strategies, and validation design, limiting direct comparison of their findings. Given that CT is not routinely used for the diagnosis of ovarian endometriosis and is instead reserved for selected acute or complex clinical scenarios, these radiomics findings should be interpreted as opportunistic and context-specific rather than indicative of a broader first-line diagnostic role for CT in this setting; their principal value likely lies in scenarios where CT is already being acquired for another clinical indication.

### 3.4. Magnetic Resonance Imaging-Based Radiomics

Two studies fulfilled the inclusion criteria and explored the diagnostic performance of MRI-based radiomics and artificial intelligence approaches for the evaluation of ovarian endometriotic cysts, with their principal characteristics and outcomes summarized in Table 4. Both investigations were conducted at single centers, comprising one retrospective and one prospective study, with sample sizes of 43 and 116 cases, respectively. The diagnostic objectives differed across studies: one focused on the differentiation of ovarian endometriomas from hemorrhagic cysts, whereas the other aimed to characterize endometriotic lesions without comparative lesion classification. MRI examinations were performed at 1.5 T and 3.0 T, using T2-weighted imaging alone in one study and a multiparametric protocol including T1-weighted, T2-weighted, and diffusion-weighted imaging in the other. Lesion delineation strategies varied, employing semi-automatic segmentation in one investigation and automatic segmentation in the other. The reference standard differed between studies: Jiang et al. used histopathological confirmation, whereas Lupean et al. [25] used a mixed reference standard combining surgical/histopathological confirmation with expert clinical and sonographic consensus for a subset of cases (see Table 4). The study addressing diagnostic classification—Lupean et al. [25]—reported an AUC of 1.00, with 100% sensitivity and specificity. The second study—Jiang et al. [46]—addressed automated lesion delineation rather than diagnostic classification; its reported detection performance (accuracy 91.3%, sensitivity ~90%, specificity 93%) reflects segmentation quality rather than diagnostic discrimination and is not directly comparable to the classification metrics reported above.

Lupean et al. [25] investigated the value of whole-lesion MRI texture analysis for differentiating ovarian endometriomas from hemorrhagic ovarian cysts, with particular emphasis on volumetric lesion characterization. Texture features were extracted from three-dimensional volumes of interest manually and semi-automatically delineated on high-resolution T2-weighted images acquired at 1.5 T. The use of whole-lesion volumetric analysis enabled extraction of texture features from the entire cystic volume rather than from a single representative image slice. Following feature selection procedures, entropy-related parameters reflecting intralesional signal variability emerged as key discriminators. A multivariable model incorporating the selected texture features achieved perfect apparent diagnostic performance, with an AUC of 1.00 and 100% sensitivity and specificity, markedly outperforming conventional MRI signs such as T2 shading and T2 dark spots. Despite these promising findings, the results should be interpreted cautiously given the small sample size, single-center design, and absence of external validation, all of which may contribute to overly optimistic estimates of diagnostic accuracy.

In contrast to handcrafted MRI texture analysis approaches, Jiang et al. [46] explored an artificial intelligence-based segmentation strategy focused on image optimization and automated lesion delineation rather than direct lesion differentiation. The authors evaluated a fuzzy C-means clustering algorithm applied to multiparametric 3.0 T MRI examinations, including T1-weighted, T2-weighted, fat-suppressed T2-weighted, and diffusion-weighted sequences. This unsupervised clustering approach was developed to improve lesion boundary delineation and reduce image noise by allowing each voxel to be assigned probabilistically to different tissue classes. Delineation performance was assessed by comparing conventional MRI interpretation, traditional hard clustering techniques, and the proposed fuzzy clustering approach, using histopathological confirmation as the reference standard for lesion presence. Whereas conventional MRI achieved an accuracy of approximately 76%, and hard clustering methods reached 85.4%, the fuzzy C-means algorithm demonstrated higher performance, with detection sensitivity of around 90%, specificity of 93%, and overall detection accuracy of 91.3%. Within this study, the fuzzy C-means approach demonstrated higher reported delineation performance than both conventional MRI interpretation and hard clustering methods, indicating the potential utility of AI-assisted image processing for automated lesion delineation—a distinct objective from diagnostic differentiation.

Collectively, the MRI-based studies investigated two distinct quantitative imaging strategies: volumetric radiomics for lesion characterization and artificial intelligence-based image processing for automated lesion segmentation. Although both studies reported strong performance on their respective tasks—diagnostic classification for Lupean et al. [25] and segmentation/delineation for Jiang et al. [46]—they addressed fundamentally different stages of the imaging workflow and are therefore not directly comparable. Furthermore, both investigations were retrospective single-center studies with limited sample sizes and without external validation. Differences in MRI acquisition protocols, segmentation methodology, feature extraction workflows, and analytical pipelines further contributed to methodological heterogeneity across the available evidence.

**Figure 5:**
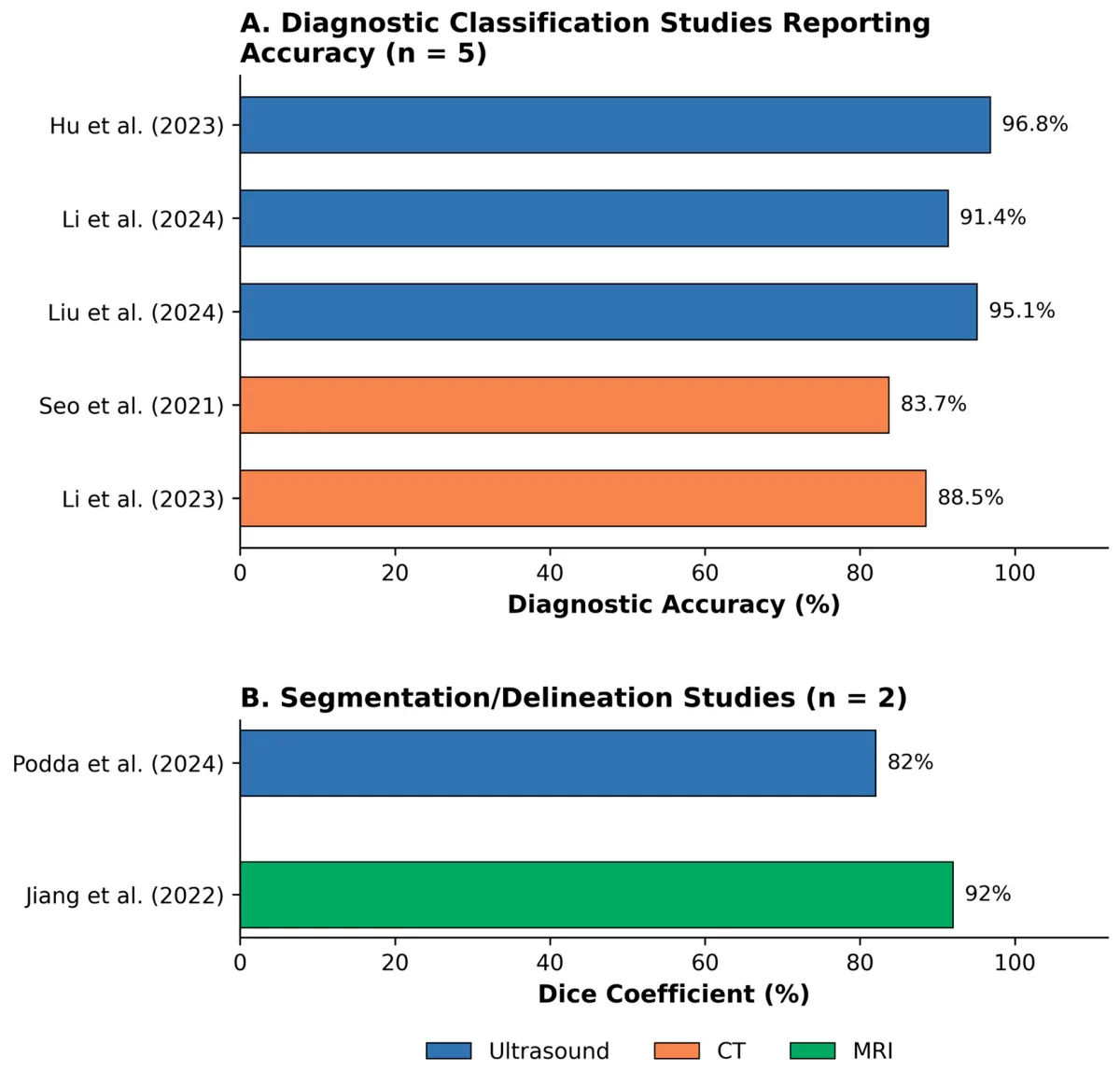
(**A**) Directly reported diagnostic accuracy for the five diagnostic-classification studies that reported this metric [40,41,42,44,45]. Ştefan et al. [39] and Lupean et al. [25] reported AUC, sensitivity, and specificity, but not accuracy, and are therefore not plotted. (**B**) Dice coefficients reported by the two studies addressing lesion segmentation/delineation, Podda et al. (2024) and Jiang et al. (2022) [43,46].

Figure 5 summarizes reported performance across the nine included studies, separated into diagnostic classification (Panel A) and segmentation/delineation (Panel B), reflecting the fundamentally different nature of these two tasks. Panel A presents directly reported accuracy for the five diagnostic-classification studies that reported this metric, ranging from 83.7% to 96.8%. Ştefan et al. [39] and Lupean et al. [25] reported AUC, sensitivity, and specificity but not accuracy directly, and are therefore not plotted; their apparent perfect discrimination (both AUC = 1.00), alongside the smallest sample sizes among all included studies, is discussed further in Section 4.3. Podda et al. [43] and Jiang et al. [46], which addressed lesion segmentation/delineation rather than diagnostic classification, are presented separately in Panel B using Dice coefficient, the metric directly reported by both studies for this task.

### 3.5. Radiomics-Specific Quality Appraisal

Beyond conventional risk-of-bias assessment (Section 3.1), methodological characteristics specific to radiomics research were systematically appraised across the nine included studies across seven domains: image preprocessing and discretization; segmentation strategy and inter-/intraobserver reproducibility; feature extraction software and IBSI compliance; whether feature selection was nested within model validation; model type, hyperparameter tuning, and leakage-prevention measures; the rigor of internal/external validation, including calibration and decision-curve analysis; and adherence to a formal reporting framework. Results are summarized in Table 5.

Full per-study methodological detail underlying this appraisal, including 95% confidence intervals, feature counts before and after selection, and validation-splitting strategy, is provided in Tables S4–S6.

This appraisal revealed considerable heterogeneity in methodological rigor across the included literature. Two studies—Li et al. [45] and Liu et al. [42]—employed IBSI-compliant feature extraction (PyRadiomics), formally assessed segmentation reproducibility via inter-observer intraclass correlation coefficients, nested feature selection within the training cohort only, and reported both calibration curves and decision-curve analysis, representing the most methodologically robust designs among the included studies. By contrast, three studies (Ştefan et al. [39], Lupean et al. [25], and Jiang et al. [46]) evaluated model performance without any independent train/test split, a design that substantially elevates the risk of overfitting and is consistent with the near-perfect diagnostic metrics reported by two of these studies (Section 3.2 and Section 3.4). Notably, none of the nine included studies reported adherence to a formal radiomics reporting framework such as TRIPOD + AI [38], CLAIM, or RQS, underscoring a broader gap in standardized methodological reporting that extends beyond this specific body of literature.

## 4. Discussion

### 4.1. Clinical Need for Improved Preoperative Characterization

Ovarian endometriosis imposes a substantial clinical and socioeconomic burden, extending beyond chronic pelvic pain to impair physical, psychological, and social well-being. Chronic pelvic pain affects approximately 71% to 90% of patients [47], whereas fatigue, sleep disturbances, and depressive symptoms are reported in 17.3–50.7% of cases, reflecting the chronic and relapsing nature of the disease [48,49]. This considerable disease burden is paralleled by high healthcare expenditures related to outpatient care, diagnostic imaging, long-term medical therapy, and surgical management, with direct medical costs estimated to range from USD 1459 to 20,239 per patient annually [50]. Moreover, indirect costs attributable to absenteeism and presenteeism account for approximately 75–84% of the total economic burden [51], corresponding to an estimated USD 4572–15,737 per patient per year and consistently exceeding direct healthcare expenditures [52]. Together, these observations emphasize the need for diagnostic strategies that facilitate more accurate, individualized, and cost-conscious clinical decision-making.

Although transvaginal ultrasound and MRI are indispensable for the detection and characterization of ovarian endometriomas, their limitations become most apparent during clinical decision-making, particularly when surgical intervention is being considered. In routine clinical practice, imaging findings are interpreted in conjunction with patient symptoms to guide management; however, this combined approach may still result in substantial diagnostic uncertainty. Notably, surgery performed for suspected endometriosis fails to confirm the diagnosis in a considerable proportion of cases. As diagnosis is still heavily reliant on laparoscopy, large retrospective cohorts have shown that up to 42.8% of women undergoing surgery for presumed endometriosis are found not to have the disease intraoperatively, and among those operated on for chronic pelvic pain, fewer than one in four (21.4%) have surgically confirmed endometriosis [53], generating an average of 6.4 years of diagnostic delay due to lack of early macroscopic changes and sampling errors [54]. These findings underscore a critical gap between imaging-based suspicion and definitive diagnosis, with important implications for patient care. In ovarian endometriosis, this uncertainty is particularly consequential, as potentially avoidable surgical interventions may expose patients to irreversible loss of ovarian reserve and compromise future reproductive potential. 

Infertility represents one of the most clinically consequential manifestations of ovarian endometriosis, affecting approximately 26% of women with the disease [55]. Women with ovarian endometriomas consistently demonstrate reduced ovarian reserve, reflected by significantly lower baseline anti-Müllerian hormone (AMH) levels and antral follicle counts compared with healthy controls. Importantly, ovarian reserve appears to decline progressively even in conservatively managed patients, with median AMH reductions of 19–29.6% within six months compared with an approximately 8% decline in women without endometriosis [56,57]. Surgical treatment may further exacerbate ovarian reserve loss, with studies reporting a median 38% decrease in AMH six months following cystectomy, particularly in patients with bilateral endometriomas or larger cysts [58]. These observations highlight the delicate balance between symptom control and fertility preservation in the management of ovarian endometriomas and emphasize the importance of accurate, noninvasive preoperative characterization to support individualized treatment decisions and minimize unnecessary surgical intervention [59].

### 4.2. Principal Findings

In the context of these clinical challenges, radiomics and deep learning have emerged as quantitative imaging approaches capable of improving the preoperative characterization of ovarian endometriotic lesions by extracting objective descriptors of tissue heterogeneity from routinely acquired imaging examinations. Across the included studies, diagnostic performance was consistently high, with several investigations reporting results comparable to, or in selected scenarios exceeding, those of experienced radiologists. For example, Li et al. [45] reported that a radiomics-based prediction model improved diagnostic accuracy by up to 16% compared with radiologist assessment, whereas Hu et al. [40] demonstrated accuracy improvements of approximately 19–32% over experienced ultrasound physicians using a deep learning-based approach. Collectively, these findings indicate that quantitative imaging analysis has the potential to improve diagnostic consistency and support lesion characterization, particularly in clinically challenging cases.

Importantly, the diagnostic contribution of radiomics appears to differ according to imaging modality and clinical objective. Ultrasound-based models represented the largest body of evidence and were primarily developed for the differential diagnosis of endometriomas from other cystic adnexal lesions, frequently demonstrating high diagnostic performance when trained on large, well-annotated datasets. CT-based approaches provided more limited but complementary evidence, suggesting improvements in reader confidence and diagnostic consistency, particularly among less experienced interpreters. Although fewer in number, MRI-based studies highlighted the potential of volumetric texture analysis and automated segmentation techniques, reflecting the superior soft-tissue contrast and multiparametric capabilities of this modality. Collectively, these findings suggest that the different imaging modalities may offer complementary rather than competing applications within the evolving radiomics landscape of ovarian endometriosis.

In the two included studies that formally combined radiomic features with clinical or biochemical variables—Liu et al. [42] and Li et al. [45] (CT)—the combined models outperformed their respective single-component comparators. Liu et al. [42] demonstrated that a nomogram combining radiomic and clinical signatures achieved diagnostic performance comparable to senior radiologists and superior to junior radiologists, with junior-radiologist accuracy improving substantially when assisted by the model. Li et al. [45] similarly found that a nomogram incorporating CA-125 and menopausal status alongside radiomic features outperformed the radiomics signature and clinical model individually in both training and validation cohorts. Hu et al. [40] evaluated an imaging-only deep-learning model and compared its performance with that of sonographers and CA-125; clinical or laboratory variables were not incorporated into the prediction model. Collectively, this pattern—drawn from the two included studies that performed a formal within-study comparison—indicates that, in these instances, combining radiomics with clinical and laboratory variables provided complementary information that enhanced diagnostic performance. A similar pattern has been reported in adjacent disease entities, including an ensemble multimodal model for deep infiltrating endometriosis [60] and a human-AI collaborative multimodal framework for endometriosis more broadly [61]; as these studies fall outside the nine ovarian endometrioma-specific studies included in this review, they are presented here as corroborating rather than primary evidence. Based primarily on the included ovarian endometrioma literature, multimodal prediction models integrating imaging, clinical, and laboratory information currently represent the most mature and clinically relevant application of radiomics identified within the evidence base of this review, though this conclusion should be interpreted as specific to the studies reviewed rather than as an established consensus across the broader endometriosis field.

### 4.3. Methodological Maturity of the Current Evidence

Despite the encouraging diagnostic performances reported in the included studies, the clinical translation of radiomics in ovarian endometriosis remains contingent on demonstrating consistent results across diverse imaging environments and patient populations [62]. Radiomics models are inherently sensitive to variations in image acquisition parameters, reconstruction techniques, and lesion segmentation strategies, factors that may introduce non-biological variability and affect feature reproducibility. In addition, differences in model development workflows and the extent of clinical data integration further limit direct comparability between studies. Consequently, studies reporting near-perfect diagnostic performance frequently rely on small datasets with internal validation only, raising concerns regarding model generalizability and potential overfitting [63]. Although integrated prediction models have shown meaningful improvements in diagnostic accuracy, their robustness must be confirmed in broader real-world settings characterized by heterogeneous imaging protocols, operator expertise, and patient characteristics. Prospective validation and methodological standardization therefore represent key prerequisites for safe and reliable clinical implementation.

Another important consideration concerns the interpretation of the exceptionally high diagnostic performance reported in several studies, including AUC values approaching 1.00 and near-perfect sensitivity and specificity. Whereas such findings may reflect strong discriminatory potential, they should be interpreted cautiously in light of methodological factors known to influence model generalizability in radiomics research. Potential contributors include feature selection performed within the same dataset used for model development, limited sample sizes relative to feature dimensionality, reliance on internal validation without independent external testing, and insufficient reporting of model calibration or clinical utility analyses [64]. Together, these factors increase the likelihood of optimistic performance estimates resulting from overfitting or subtle forms of data leakage, particularly in retrospective single-center studies. This pattern is particularly evident when the number of extracted features is considered relative to sample size: Ștefan et al. [39] and Lupean et al. [25]—the two studies reporting perfect apparent discrimination (AUC = 1.00)—had feature-to-patient ratios of approximately 4.91:1 and 7.95:1, respectively, both well above conventional stability thresholds for radiomics feature selection. Notably, Li et al. [45] also reported a high feature-to-patient ratio of approximately 4.59:1—although lower than the ratios reported by Ştefan et al. and Lupean et al.—without achieving perfect classification (AUC = 0.942), suggesting that whilst a high feature-to-patient ratio increases the risk of overfitting, it is not the sole determinant of inflated performance estimates; model development choices, validation strategy, and feature selection methodology likely also contribute.

This risk is compounded in ultrasound-based studies, where residual boundary distortion from speckle noise and acoustic artifacts can inflate the correlation structure among extracted texture features, further increasing the dimensionality burden relative to sample size [65,66]. Consequently, robust feature selection and regularization strategies are not merely beneficial but necessary to control redundancy and mitigate model instability. This concern extends to the integrated clinical-radiomic nomograms reported in several included studies [42], where combining radiomic signatures with clinical variables further increases the effective parameter space without a corresponding increase in sample size, warranting particular caution in interpreting their apparent performance gains. In contrast, studies incorporating independent test cohorts or integrating clinical variables into multivariable prediction models generally reported more conservative, yet potentially more clinically interpretable, performance estimates.

Beyond issues of reproducibility, the current body of evidence is characterized by substantial methodological heterogeneity. Included studies differed markedly in imaging modality (ultrasound, CT, MRI), analytical objectives (lesion segmentation versus diagnostic classification), radiomics paradigms (handcrafted feature extraction versus deep learning), dimensional representation of lesions (two-dimensional versus volumetric analysis), and validation strategies [67]. As detailed in the radiomics-specific quality appraisal (Table 5), reporting of segmentation reproducibility and feature normalization procedures was inconsistent across studies, and none of the nine included studies reported adherence to a formal radiomics or artificial intelligence reporting framework, including IBSI-aligned feature extraction or TRIPOD + AI principles [36,38], further limiting comparability across studies. Although such diversity reflects the exploratory stage of radiomics research in ovarian endometriosis, it complicates cross-study comparison, limits the ability to draw modality-specific or technique-specific clinical conclusions, and hinders meaningful evidence synthesis. Harmonized methodological frameworks and standardized reporting will therefore be essential to improve comparability across studies and facilitate future clinical translation.

Overall, the available evidence indicates that radiomics in ovarian endometriosis remain at an early stage of methodological maturity. Although the reported diagnostic performance is encouraging, the current evidence should be regarded as preliminary and hypothesis-generating, with meaningful clinical translation dependent on standardized analytical pipelines, multicenter external validation, and improved reporting transparency.

### 4.4. Readiness for Clinical Translation

From a practical perspective, one of the main advantages of radiomics-based approaches is that they build upon imaging examinations already routinely performed during the diagnostic evaluation of suspected ovarian endometriosis, particularly transvaginal ultrasound and MRI. Consequently, their implementation does not inherently require additional invasive procedures or dedicated patient interventions. When integrated into clinically accessible software platforms, radiomics-based tools could facilitate semi-automated lesion assessment, improve diagnostic reproducibility, and support more standardized, risk-adapted clinical decision-making. Although current radiomics workflows remain largely confined to research settings and continue to lack methodological standardization, their integration into existing imaging workflows represents a realistic pathway toward scalable imaging-based decision-support systems [68].

Beyond statistical performance, the increasing use of integrated radiomics-based prediction models raises important questions regarding the biological relevance of imaging-derived features and their relationship with established laboratory biomarkers. Radiomic features reflecting lesion heterogeneity and internal signal distribution may indirectly capture pathological processes characteristic of ovarian endometriosis, including cyclic hemorrhage, fibrotic remodeling, and chronic inflammatory activity [69]. These processes are also reflected, albeit through different biological pathways, in circulating biomarkers such as CA-125, which primarily reflects inflammatory burden rather than disease-specific histopathology [40]. Consequently, combining radiomics with clinical and laboratory variables may provide complementary information that enables a more comprehensive characterization of disease phenotype than either approach alone, supporting the development of multimodal diagnostic frameworks.

From a clinical perspective, the role of radiomics in ovarian endometriosis should be interpreted within the context of existing high-performing diagnostic approaches, particularly expert transvaginal ultrasound. Radiomics is unlikely to replace established imaging criteria in typical cases; rather, its greatest potential lies in selected clinical scenarios characterized by diagnostic uncertainty, such as atypical cyst morphology, overlapping imaging features, or limited operator expertise [70]. In these settings, radiomics-based decision-support models integrating quantitative imaging features with clinical and laboratory variables may provide additional objective information to support lesion characterization, improve diagnostic confidence, and contribute to more individualized management strategies. However, current evidence does not yet demonstrate measurable improvements in patient-important outcomes, reduction in unnecessary interventions, or cost-effectiveness, and its implementation in routine clinical practice remains constrained by methodological variability and the lack of standardized workflows [71]. At present, radiomics should therefore be regarded as an exploratory adjunct to expert image interpretation, with broader clinical adoption dependent on prospective multicenter validation and demonstration of meaningful added value beyond current diagnostic practice.

Translating these findings into practice requires clarity on both when radiomics is likely to add value and what stands in the way of its adoption. Radiomics-based tools are most plausibly useful in scenarios where diagnostic uncertainty persists after conventional imaging—for example, atypical adnexal cyst morphology, discordant clinical and sonographic findings, evaluation by less experienced operators without ready access to expert second review, or acute and complicated presentations such as spontaneous rupture of an ovarian endometrioma—rather than as a replacement for expert image interpretation in straightforward cases. In such acute settings, CT is often the first modality acquired in the emergency department and can facilitate rapid recognition of a pelvic mass and associated hemoperitoneum, whereas MRI is typically required to refine the differential diagnosis, exclude mimickers such as tubo-ovarian abscess, mature cystic teratoma, or malignancy, and support preoperative planning [72]. This sequential, complementary use of CT and MRI in emergency and complex presentations further underscores that CT-based radiomics is best conceptualized as an opportunistic adjunct in selected clinical scenarios rather than a candidate for routine first-line endometrioma assessment.

Practical implementation, however, faces several concrete barriers. First, most radiomics pipelines remain vendor- and protocol-specific, with feature values sensitive to acquisition parameters, reconstruction settings, and segmentation method, limiting portability across imaging systems and institutions. Second, clinical integration requires validated, regulatory-cleared software embedded within existing PACS (Picture Archiving and Communication System) and reporting workflows, which remains largely absent for ovarian endometriosis-specific applications. Third, as noted above, the absence of outcome-level or cost-effectiveness data makes it difficult to justify adoption to clinical stakeholders or payers in the near term. Finally, routine use would require standardized training for radiologists and technologists and mechanisms for ongoing quality assurance, both of which are currently undefined in this field. A related barrier concerns model interpretability: several of the included deep-learning-based studies rely on architectures whose internal decision-making is not readily interpretable by clinicians, limiting trust and accountability in a diagnostic context. The integration of explainable AI (XAI) techniques—such as saliency maps, feature attribution methods, or model-agnostic explanation frameworks—has not yet been explored in this domain and represents an important direction for future work aiming to translate radiomics- and AI-based tools into clinically trusted decision-support systems. Deep-learning-based approaches additionally require substantial computational resources for model training, validation, and testing to ensure generalizability; reported processing times for automated pipelines in comparable segmentation tasks range from under 3 min to over 40 min depending on the method and computing infrastructure used [73,74,75], which may limit accessibility for institutions without dedicated computing resources. Addressing these barriers, alongside the methodological standardization discussed above, represents a necessary precondition for moving radiomics from a research tool to a clinically deployable adjunct [19,76].

## 5. Limitations

Several limitations should be considered when interpreting the findings of this review. Most included studies were retrospective, single-center investigations with relatively small sample sizes, which may limit the robustness and generalizability of the reported findings. In addition, substantial methodological heterogeneity was observed across studies, including differences in imaging modalities, acquisition protocols, segmentation methods, feature extraction pipelines, machine learning algorithms, and validation strategies, limiting direct comparison between studies. Additionally, the search was limited to PubMed, Scopus, and Web of Science; databases with a stronger engineering and computer-science orientation, such as IEEE Xplore, were not included and may contain additional technically oriented studies not indexed in the databases searched. External validation using independent datasets was uncommon, increasing the risk of model overfitting and optimistic performance estimates. Finally, the incorporation of clinical and laboratory variables into radiomics models was inconsistent, and, as detailed in the radiomics-specific quality appraisal (Section 3.5, Table 5), none of the included studies reported adherence to a standardized framework for model development, validation, or reporting, such as TRIPOD + AI [38], CLAIM, or RQS. Publication bias should also be considered, as studies reporting high diagnostic performance may be preferentially submitted and accepted for publication, whereas studies with modest or negative findings may remain unpublished; given the small number of included studies, a formal quantitative assessment of publication bias (e.g., funnel plot analysis) was not feasible, limiting our ability to evaluate this risk directly. In addition, none of the included studies reported cost-effectiveness or economic feasibility data, precluding any assessment of the resource implications of implementing radiomics-based tools in clinical workflows. Finally, all included studies were conducted in single, high-volume academic or tertiary referral centers with subspecialized imaging expertise; the generalizability of these findings to non-specialist or community-based settings, where operator experience, equipment quality, and disease case-mix may differ substantially, therefore remains uncertain. Collectively, these limitations emphasize the need for larger prospective multicenter studies employing standardized radiomics methodologies and rigorous external validation before widespread clinical implementation can be considered.

## 6. Conclusions

Radiomics represents an emerging extension of conventional imaging for the evaluation of ovarian endometriosis, providing objective quantitative information that may complement lesion characterization and preoperative decision-making. The current evidence suggests that its greatest clinical value lies not in replacing established imaging modalities, but in supporting diagnostic assessment through integration with clinical and laboratory variables within multivariable prediction models. Although the reported diagnostic performance is encouraging, the available evidence remains methodologically heterogeneous and is largely derived from retrospective single-center studies with limited external validation. Consequently, broader clinical implementation will require standardized radiomics workflows, rigorous prospective multicenter validation, and demonstration of meaningful added value beyond current diagnostic practice. As these challenges are addressed, radiomics has the potential to become a valuable adjunct to expert imaging in selected patients with ovarian endometriosis. Future work could also usefully extend beyond the AI/radiomics-specific scope of the present review to include conventional (non-AI) radiomics pipelines and studies incorporating healthy-control comparisons, which may offer additional insight into processing methodology and the distinguishing imaging characteristics of pathological versus healthy ovarian tissue.

## Figures and Tables

**Table 1:** Comparison of the present review with prior narrative reviews on ovarian endometriosis.

Feature	Busacca & Vignali, 2003 [29]	Psaroudakis et al., 2014 [30]	Arafah et al., 2021 [31]	Mikhaleva et al., 2020 [32]	Giannini et al., 2024 [33]	Nezhat et al., 2008 [34]	This Review
Covers ovarian endometriosis	✓	✓	✓	✓	✓	✓	✓
Discusses imaging diagnosis	–	–	–	–	–	–	✓
Covers radiomics/quantitative imaging features	–	–	–	–	–	–	✓
Covers AI/machine learning applications	–	–	–	–	–	–	✓
Systematic methodology (PRISMA)	–	–	–	–	–	–	✓
Formal multi-database search strategy	–	–	–	–	–	–	✓
Diagnostic performance synthesis	–	–	–	–	–	–	✓
Addresses pathogenesis/malignant transformation	✓	–	✓	✓	✓	✓	–
Addresses surgical/medical management	✓	✓	–	–	–	–	–

AI = Artificial intelligence; PRISMA = Preferred Reporting Items for Systematic Reviews and Meta-Analyses; ✓ = feature present; – = feature absent.

**Table 2:** Overview of included ultrasound-based radiomics studies for the characterization of ovarian endometriosis.

No.	Author, Year	Study Design	Sample Size	Classification Task	US Technique	Segmentation	Extracted Features/ Model Type	Feature-to-Patient Ratio	Reference Standard	Validation	Performance
1.	Ștefan et al., 2021 [39]	Retrospective Single center	56	Diagnostic classification Ovarian endometriomas versus hemorrhagic ovarian cysts (30 versus 26)	TVUS 4–10 MHz intracavitary probe	Semi-automatic 2D ROI segmentation with manual adjustment	First- and second-order features *n* = 275	4.91:1	Cystectomy Oophorectomy	Internal validation only	AUC = 1 Se = 100% Sp = 100% Acc = NR
2.	Hu et al., 2023 [40]	Retrospective Single center	202 Training *n* = 171 Testing *n* = 31	Diagnostic classification Ovarian endometriomas versus tubal–ovarian abscess (120 versus 82)	TVUS and transabdominal US 3.5–6.0 MHz convex array probes 7.5–12 MHz intracavitary probes	Manual	First- and second-order features *n* = NR Deep-learning models: ResNet-152, DenseNet-161, and EfficientNet-B7	NR	Cystectomy Oophorectomy	Internal validation and independent test cohort	AUC = 0.986 Se = 90% Sp = 100% Acc = 96.8%
3.	Li et al., 2024 [41]	Retrospective Single center	406	Diagnostic classification Ovarian endometriomas versus non-endometrioma cysts (211 versus 195)	TVUS and transabdominal US Probe frequency NR	Automatic	First- and second-order features *n* = NR Deep-learning models: ResNet-50	NR	Expert clinical and sonographic consensus	Five-fold cross-validation	AUC = NR Se = 95.36% Sp = 87.15% Acc = 91.36%
4.	Liu et al., 2024 [42]	Retrospective Single center	407	Diagnostic classification Ovarian endometriomas versus dermoid cysts (200 versus 207)	TVUS and TRUS 4–9 MHz intracavitary probes	Manual	First- and second-order features *n* = 107 Machine-learning models: LR, SVM, kNN, LightGBM, MLP	0.26:1	Mixed: Surgical lesion resection or Expert clinical and sonographic consensus	Internal split 80–20%	AUC = 0.967 Se = 87.9% Sp = 97.1% Acc = 92.6%
5.	Podda et al., 2024 [43]	Retrospective Single center	75	Segmentation/delineation Segmentation of ovarian endometriotic lesions	TVUS Probe frequency NR	Manual	First- and second-order features *n* = NR Deep-learning models: DenseNet121 U-Net, VGG19 U-Net, Custom U-Net	NR	Prior clinical diagnosis of endometriosis	Internal split 80–20% Five-fold cross-validation	AUC = NR Se = 78.5% Sp = 90.6% Dice = 82% Jaccard = 71%

US = ultrasound; TVUS = transvaginal ultrasound; TRUS = transrectal ultrasound; MHz = megahertz; ROI = region of interest; AUC = area under the curve; Se = sensitivity; Sp = specificity; Acc = accuracy; NR = not reported; LR = Logistic Regression; SVM = Support Vector Machine; kNN = k-Nearest Neighbors; MLP = Multilayer Perceptron. Note 1: For studies addressing segmentation/delineation rather than diagnostic classification, reported sensitivity, specificity, and accuracy reflect lesion detection/delineation performance and are not directly comparable to the diagnostic classification metrics reported for the other studies in this table. Note 2: The study reporting perfect apparent diagnostic performance (AUC = 1.00; Ştefan et al.) had the smallest sample size among the diagnostic classification studies in this table and relied on internal validation only, which may result in overoptimistic performance estimates (see Section 4.3).

**Table 3:** Overview of included computed tomography-based radiomics studies for the characterization of ovarian endometriosis.

No.	Author, Year	Study Design	Sample Size	Classification Task	CT Acquisition Protocol	Segmentation	Extracted Features/Model Type	Feature-to-Patient Ratio	Reference Standard	Validation	Performance
1.	Seo et al., 2021 [44]	Retrospective Single center	135	Diagnostic classification Ovarian endometriomas versus benign cysts (43 versus 92)	Contrast-enhanced CT Portal venous phase 5 mm slice thickness	Manual	First-order features *n* = 42	0.31:1	Cystectomy Surgical lesion resection	Internal validation only	AUC = NR Se = 72.7% Sp = 89% Acc = 83.7%
2.	Li et al., 2023 [45]	Retrospective Single center	287 Training *n* = 200 Testing *n* = 87	Diagnostic classification Ovarian endometriomas versus cystadenomas (91 versus 196)	Contrast-enhanced CT Portal venous phase 1–2.5 mm slice thickness	Manual	First- and second-order features Filtered features *n* = 1316	4.59:1	Cystectomy Oophorectomy	Internal split 70–30%	AUC = 0.942 Se = 90% Sp = 87.7% Acc = 88.5%

CT = Computed Tomography; AUC = area under the curve; Se = sensitivity; Sp = specificity; Acc = accuracy; NR = not reported.

**Table 4:** Overview of included magnetic resonance imaging-based radiomics studies for the characterization of ovarian endometriosis.

No.	Author, Year	Study Design	Sample Size	Classification Task	MRI Acquisition Protocol	Segmentation	Extracted Features/Model Type	Feature-to-Patient Ratio	Reference Standard	Validation	Performance
1.	Lupean et al., 2020 [25]	Retrospective Single center	43	Diagnostic classification Ovarian endometriomas versus hemorrhagic ovarian cysts (29 versus 14)	1.5 Tesla T2WI used for analysis	Semi-automatic 3D VOI segmentation with manual adjustment	First- and second-order features *n* = 342 Multivariate logistic regression	7.95:1	Mixed: Surgical lesion resection or Expert clinical and sonographic consensus	Internal validation only	AUC = 1 Se = 100% Sp = 100% Acc = NR
2.	Jiang et al., 2022 [46]	Prospective Single center	116	Segmentation/delineation Segmentation of ovarian endometriotic lesions	3 Tesla T1WI, T2WI and DWI used for analysis	Automatic	First- and second-order features *n* = NR Improved fuzzy C-means clustering algorithm	NR	Cystectomy Oophorectomy	Internal validation only	AUC = 0.943 Se = 90% Sp = 93% Acc = 91.31%

MRI = Magnetic Resonance Imaging; T1WI = T1-weighted imaging; T2WI = T2-weighted imaging; DWI = diffusion-weighted imaging; VOI = volume of interest; AUC = area under the curve; Se = sensitivity; Sp = specificity; Acc = accuracy; NR = not reported. Note 1: For studies addressing segmentation/delineation rather than diagnostic classification, reported sensitivity, specificity, and accuracy reflect lesion detection/delineation performance and are not directly comparable to the diagnostic classification metrics reported for the other studies in this table. Note 2: The study reporting perfect apparent diagnostic performance (AUC = 1.00; Lupean et al.) had the smallest sample size among all included studies and relied on internal validation only, which may result in overoptimistic performance estimates (see Section 4.3).

**Table 5:** Radiomics-specific quality appraisal across the 9 included studies.

Study (Modality, Task)	Preprocessing/ Resampling/Normalization/ Discretization	Segmentation Strategy + Inter/Intra-Observer Reproducibility	Feature Extraction Software + IBSI Compliance	Feature Selection Nested in Validation?	Model Type/Hyperparameter Tuning/ Leakage Prevention	Internal/ External Validation/ Calibration/DCA	Reporting Framework
Ştefan (US, diagnostic) [39]	Topaz DeNoise AI denoising; no discretization/bin-width detail reported	Not assessed/not reported	MaZda; no IBSI compliance	Not nested—3-method selection (MI/Fisher/POE + ACC) + ICC filter + Bonferroni + VIF on whole dataset	Multivariable logistic regression; no train/test split—high leakage risk	No train/test split; internal same-dataset evaluation only; no calibration; no DCA	None
Hu (US, diagnostic) [40]	Resize to 224 × 224 + augmentation; N/A (raw CNN)	N/A (deep learning); not reported	N/A—deep features, not handcrafted	N/A—transfer learning	3 CNNs (ResNet-152, DenseNet-161, EfficientNet-B7); multi-image-per-patient averaging reduces leakage	Genuine patient-level temporal external test cohort (171 vs. 31)—real strength; no calibration; no DCA	None
Li 2024 (US, multi-task) [41]	Letterbox resize to 512 × 512 + augmentation; N/A	Swin Transformer + Upernet branch; senior-expert annotation; reproducibility not reported	N/A—deep features via CA module	N/A—end-to-end DL	Hybrid CNN–Transformer, staged training; patient-level train/test overlap not addressed	5-fold CV + same-distribution hold-out (not external); reader study vs. 5 junior + 2 senior radiologists; no calibration; no DCA	None
Liu 2024 (US, diagnostic) * [42]	PyRadiomics-based extraction	ITK-SNAP, two independent radiologists, ICC ≥ 0.75	PyRadiomics, IBSI-aligned; 107 features	Properly nested—Mann–Whitney U + Spearman filter + LASSO (10-fold CV), training cohort only	Multiple ML classifiers (LR/SVM/KNN/LightGBM/MLP); 5-fold CV tuning; genuine 326/81 patient-level split	DeLong test; calibration + Hosmer-Lemeshow; DCA; reader study w/AI-assisted junior arm	None named, but comprehensive
Seo (CT, diagnostic) [44]	TexRAD; no IBSI; discretization not detailed	Genuine interobserver test (2 readers, 135 pts), no held-out cohort	TexRAD; no IBSI	Not clearly nested—trees built on Reader 1, applied to Reader 2	Decision-tree models; tuning not clearly reported	No held-out cohort; no calibration; no DCA	None
Li 2023 (CT, diagnostic) * [45]	PyRadiomics, IBSI-aligned; resampling 1 × 1 × 1 mm^3^ + bin width 25HU	Formal ICC on 30-pt subsample (intra + inter)	PyRadiomics, IBSI-aligned	Properly nested—LASSO on training cohort only	Genuine patient-level split (200/87)	Calibration curves; DCA	None named, but the standout of the 9
Lupean (MRI, diagnostic) [25]	MaZda; no IBSI	Authors explicitly state reproducibility not assessed	MaZda; no IBSI	Not nested—whole *n* = 43 used for selection + eval	Severe multicollinearity (VIF up to 3669), unaddressed	Explicitly no train/test split; no calibration; no DCA	None
Podda (US, segmentation) [43]	Multi-scale resize (64–256 px) + Albumentation augmentation	Hasty tool, expert operators; reproducibility not reported	N/A—pixel-wise DL segmentation	N/A	Multi-scale U-net ensemble (DenseNet121/VGG19/custom), fusion strategy; hyperparameters specified	Internal-only 5-fold CV, 80/20 split; no external cohort; no calibration; no DCA	None
Jiang (MRI, segmentation) [46]	No standardization beyond clustering algorithm	No comparator lesion group; FCM vs. HCM baseline	Conventional clustering-based image segmentation without radiomic feature extraction	N/A	FCM clustering vs. HCM; no train/test split	No train/test split; no calibration; no DCA	None

* = the two studies with the strongest methodological safeguards (Li et al., 2023 [45] and Liu et al., 2024 [42]).

## Data Availability

The original contributions presented in this study are included in the article/Supplementary Material. Further inquiries can be directed to the corresponding author.

## Supplementary Materials

The following supporting information can be downloaded at: https://www.mdpi.com/article/10.3390/bioengineering13080855/s1. Table S1: Complete electronic search strategies for PubMed, Scopus, and Web of Science; Table S2: Complete PRISMA 2020 checklist; Table S3: Detailed domain-level judgments and the rationale for each QUADAS-2 assessment; Table S4: Extended per-study methodological detail for the ultrasound-based radiomics studies included in this review, supplementing Table S2; Table S5: Extended per-study methodological detail for the computed tomography-based radiomics studies included in this review, supplementing Table S3; Table S6: Extended per-study methodological detail for the magnetic resonance imaging-based radiomics studies included in this review, supplementing Table S4.
