# Supplementary material for: Radiomics and Artificial Intelligence in Ovarian Endometriosis Imaging: A Systematic Review and Critical Appraisal of Emerging Evidence
Source: Bioengineering (Basel). 2026 Jul 24;13(8):855. doi: 10.3390/bioengineering13080855 (PMC13509516; doi:10.3390/bioengineering13080855)
Supplement: Supplementary file 1 [file bioengineering-13-00855-s001.zip › Figure 1.pdf]

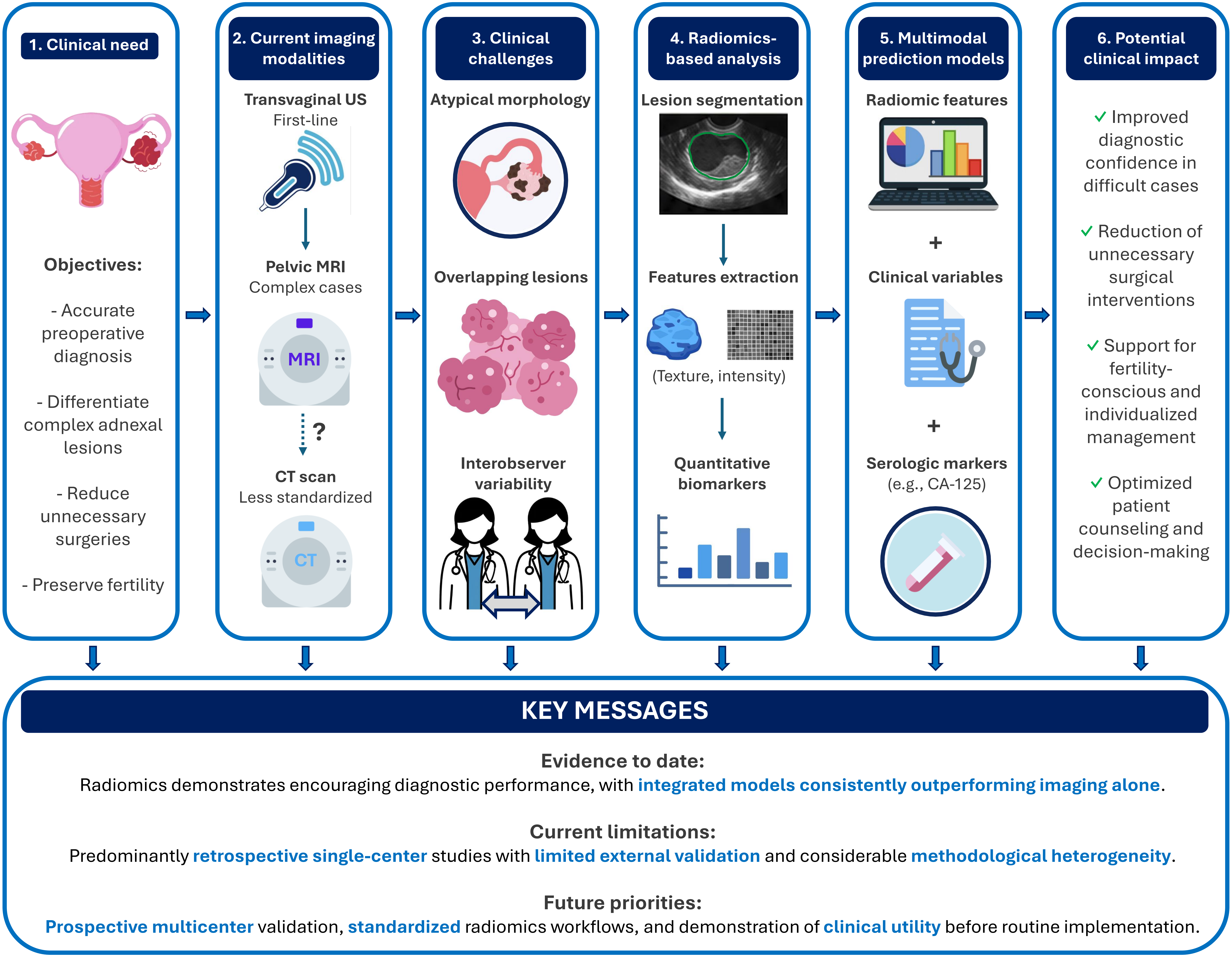

1. Clinical need
Objectives:
- Accurate preoperative diagnosis
- Differentiate complex adnexal lesions
- Reduce unnecessary surgeries
- Preserve fertility
2. Current imaging modalities
Transvaginal US
First-line
Pelvic MRI
Complex cases
MRI
?
CT scan
Less standardized
CT
3. Clinical challenges
Atypical morphology
Overlapping lesions
Interobserver variability
4. Radiomics-based analysis
Lesion segmentation
Features extraction
(Texture, intensity)
Quantitative biomarkers
5. Multimodal prediction models
Radiomic features
+
Clinical variables
+
Serologic markers
(e.g., CA-125)
6. Potential clinical impact
✓ Improved diagnostic confidence in difficult cases
✓ Reduction of unnecessary surgical interventions
✓ Support for fertility-conscious and individualized management
✓ Optimized patient counseling and decision-making
KEY MESSAGES
Evidence to date:
Radiomics demonstrates encouraging diagnostic performance, with integrated models consistently outperforming imaging alone.
Current limitations:
Predominantly retrospective single-center studies with limited external validation and considerable methodological heterogeneity.
Future priorities:
Prospective multicenter validation, standardized radiomics workflows, and demonstration of clinical utility before routine implementation.
