# Supplementary material for: Radiomics and Artificial Intelligence in Ovarian Endometriosis Imaging: A Systematic Review and Critical Appraisal of Emerging Evidence
Source: Bioengineering (Basel). 2026 Jul 24;13(8):855. doi: 10.3390/bioengineering13080855 (PMC13509516; doi:10.3390/bioengineering13080855)
Supplement: Supplementary file 1 [file bioengineering-13-00855-s001.zip › Figure 2_BRACKETS.pdf]

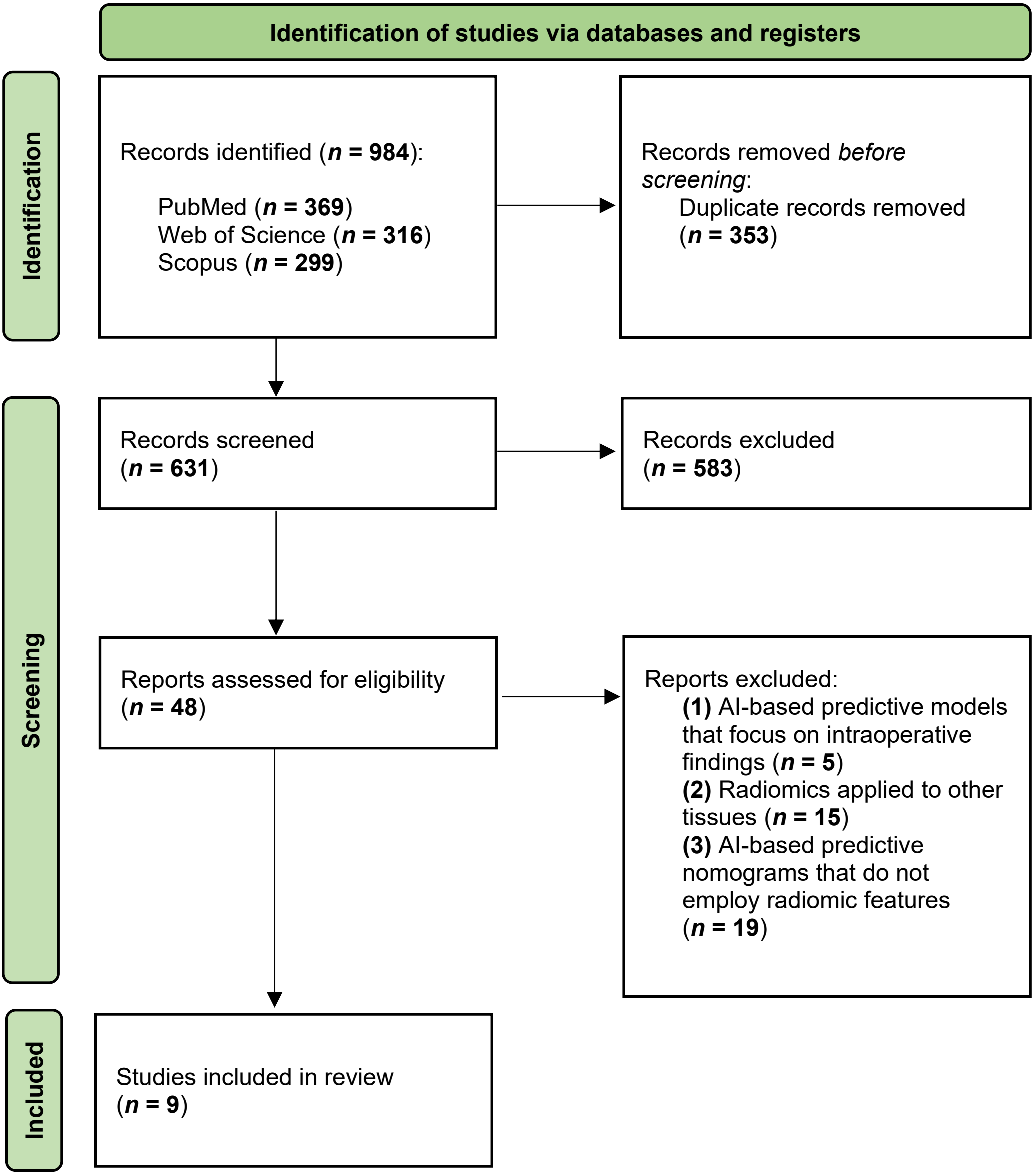
Identification of studies via databases and registers
Identification
Records identified (n = 984):
PubMed (n = 369)
Web of Science (n = 316)
Scopus (n = 299)
Records removed before screening:
Duplicate records removed (n = 353)
Screening
Records screened (n = 631)
Records excluded (n = 583)
Reports assessed for eligibility (n = 48)
Reports excluded:
(1) AI-based predictive models that focus on intraoperative findings (n = 5)
(2) Radiomics applied to other tissues (n = 15)
(3) AI-based predictive nomograms that do not employ radiomic features (n = 19)
Included
Studies included in review (n = 9)
