# Supplementary material for: Radiomics and Artificial Intelligence in Ovarian Endometriosis Imaging: A Systematic Review and Critical Appraisal of Emerging Evidence
Source: Bioengineering (Basel). 2026 Jul 24;13(8):855. doi: 10.3390/bioengineering13080855 (PMC13509516; doi:10.3390/bioengineering13080855)
Supplement: Supplementary file 1 [file bioengineering-13-00855-s001.zip › Figure 3.pdf]

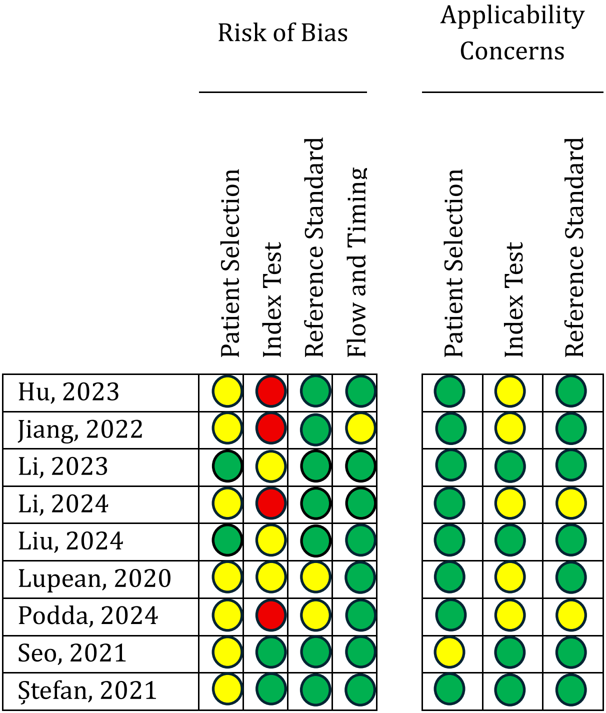
Risk of Bias
Applicability Concerns
Patient Selection
Index Test
Reference Standard
Flow and Timing
Patient Selection
Index Test
Reference Standard
Hu, 2023
Jiang, 2022
Li, 2023
Li, 2024
Liu, 2024
Lupean, 2020
Podda, 2024
Seo, 2021
Ștefan, 2021
