# Supplementary material for: Radiomics and Artificial Intelligence in Ovarian Endometriosis Imaging: A Systematic Review and Critical Appraisal of Emerging Evidence
Source: Bioengineering (Basel). 2026 Jul 24;13(8):855. doi: 10.3390/bioengineering13080855 (PMC13509516; doi:10.3390/bioengineering13080855)
Supplement: Supplementary file 1 [file bioengineering-13-00855-s001.zip › Figure 4.pdf]

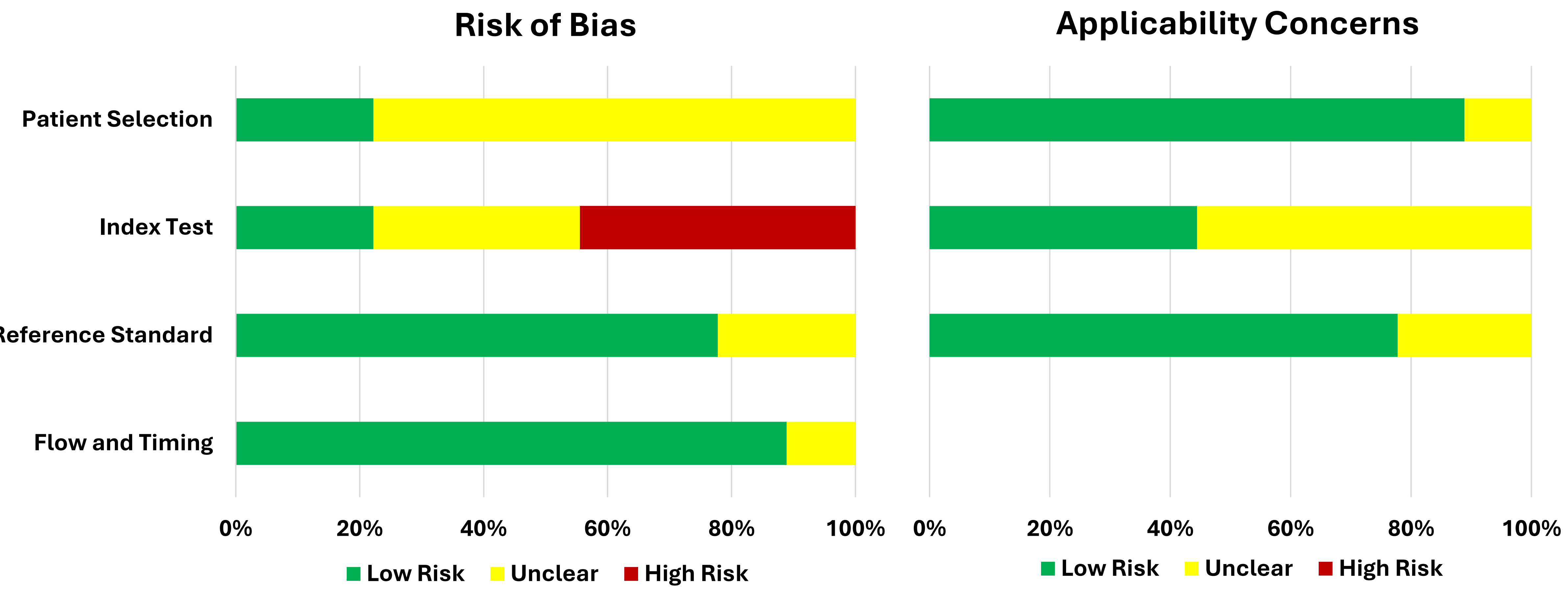
Risk of Bias
Applicability Concerns
Patient Selection
Index Test
Reference Standard
Flow and Timing
0%
20%
40%
60%
80%
100%
Low Risk
Unclear
High Risk
