# Supplementary material for: Radiomics and Artificial Intelligence in Ovarian Endometriosis Imaging: A Systematic Review and Critical Appraisal of Emerging Evidence
Source: Bioengineering (Basel). 2026 Jul 24;13(8):855. doi: 10.3390/bioengineering13080855 (PMC13509516; doi:10.3390/bioengineering13080855)
Supplement: Supplementary file 1 [file bioengineering-13-00855-s001.zip › Figure 5_corrected_v4.pdf]

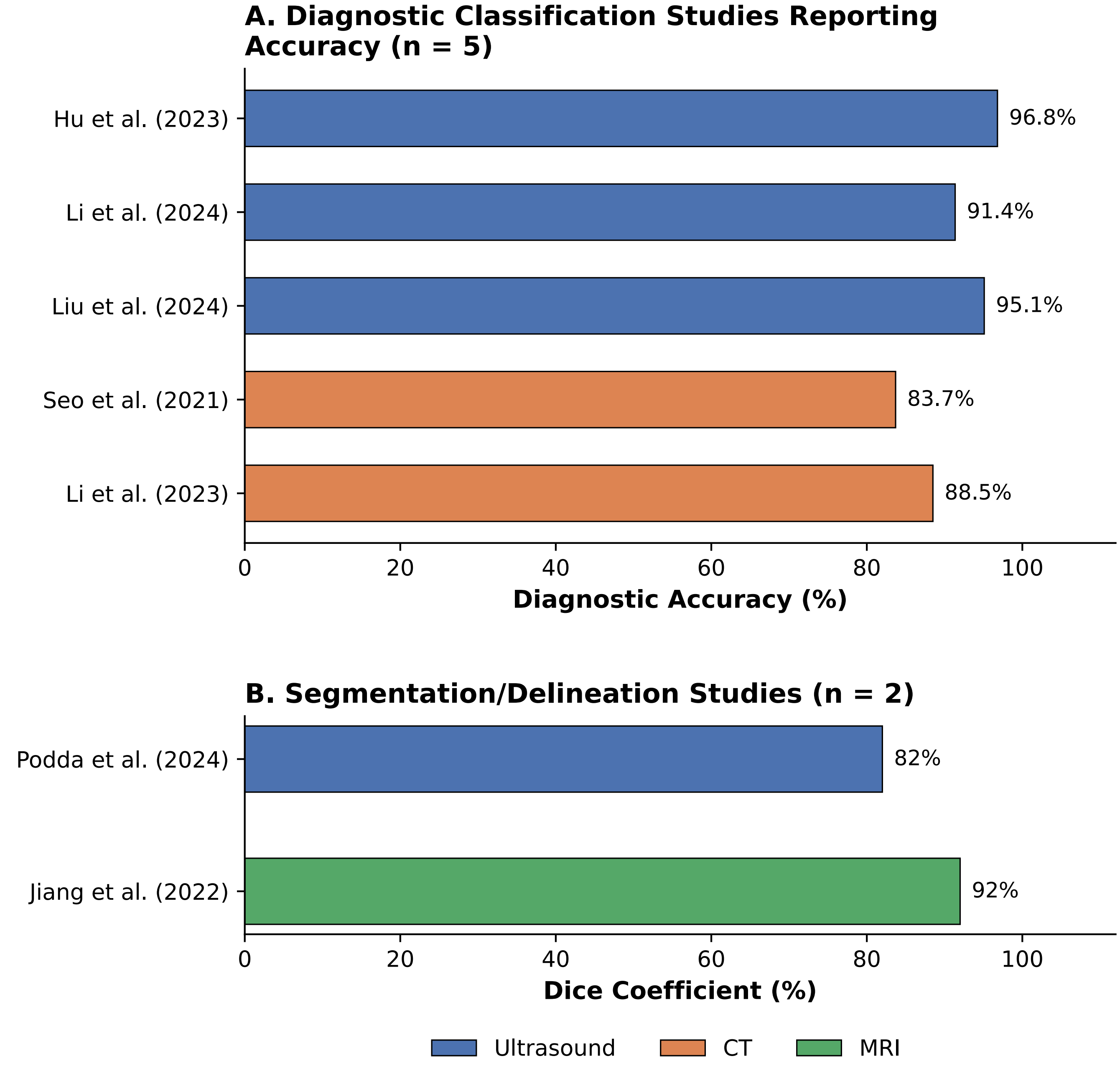
A. Diagnostic Classification Studies Reporting Accuracy (n = 5)
Hu et al. (2023)
96.8%
Li et al. (2024)
91.4%
Liu et al. (2024)
95.1%
Seo et al. (2021)
83.7%
Li et al. (2023)
88.5%
0
20
40
60
80
100
Diagnostic Accuracy (%)
B. Segmentation/Delineation Studies (n = 2)
Podda et al. (2024)
82%
Jiang et al. (2022)
92%
0
20
40
60
80
100
Dice Coefficient (%)
Ultrasound
CT
MRI
