# Supplementary material for: Radiomics and Artificial Intelligence in Ovarian Endometriosis Imaging: A Systematic Review and Critical Appraisal of Emerging Evidence
Source: Bioengineering (Basel). 2026 Jul 24;13(8):855. doi: 10.3390/bioengineering13080855 (PMC13509516; doi:10.3390/bioengineering13080855)
Supplement: Supplementary file 1 [file bioengineering-13-00855-s001.zip › Supplementary File 1.pdf]

Database	Search date	Search fields	Complete search strategy	Filters/limits applied	Records retrieved
**PubMed**	28 Mar 2026	MeSH, Title/Abstract	(("Endometriosis"[Mesh] OR "ovarian endometriosis" OR endometrioma OR "endometrioid cyst")) AND (("Radiomics"[Mesh] OR radiomic* OR "texture analysis" OR "machine learning" OR "deep learning" OR "artificial intelligence" OR "computer-assisted diagnosis" OR CAD))	English; No publication date restriction; No study design filters	369
**Scopus**	29 Mar 2026	TITLE-ABS-KEY	TITLE-ABS-KEY("ovarian endometriosis" OR endometrioma OR "endometrioid cyst") AND TITLE-ABS-KEY(radiomic* OR "texture analysis" OR "machine learning" OR "deep learning" OR "artificial intelligence" OR "computer assisted diagnosis" OR CAD)	English; No publication date restriction; No study design filters	299
**Web of Science**	31 Mar 2026	Topic (TS)	TS=("ovarian endometriosis" OR endometrioma OR "endometrioid cyst") AND TS=(radiomic* OR "texture analysis" OR "machine learning" OR "deep learning" OR "artificial intelligence" OR "computer assisted diagnosis" OR CAD)	English; No publication date restriction; No study design filters	316

Table S1. Complete electronic search strategies for PubMed, Scopus, and Web of Science.

** A post-hoc sensitivity analysis using the wildcard endom* (identifying 6,448 records vs. 984 with the registered strategy) was conducted in response to peer review. The wildcard search identified 250 candidate records after deduplication and restriction to records containing both an ovary-related and an endometriosis-related term. After restricting these to records containing the narrower endometrio*-family stem (endometriosis, endometrioma, endometriotic, or endometrioid) rather than the broader endometri* stem — which also matches unrelated terms such as endometrium, endometrial, and endometritis — 122*

records remained for manual eligibility screening; no additional eligible studies were identified (full details available from the corresponding author upon request).
