# Supplementary material for: Radiomics and Artificial Intelligence in Ovarian Endometriosis Imaging: A Systematic Review and Critical Appraisal of Emerging Evidence
Source: Bioengineering (Basel). 2026 Jul 24;13(8):855. doi: 10.3390/bioengineering13080855 (PMC13509516; doi:10.3390/bioengineering13080855)
Supplement: Supplementary file 1 [file bioengineering-13-00855-s001.zip › Supplementary File 2.pdf]

PRISMA

PRISMA 2020 Checklist

Section and Topic	Item #	Checklist item	Location where item is reported
**TITLE**			
Title	1	Identify the report as a systematic review.	**Title page**<br>Identifies the report as a systematic review
**ABSTRACT**			
Abstract	2	See the PRISMA 2020 for Abstracts checklist.	**Abstract,**<br>Structured abstract summarizing background, objective, methods, results, and conclusions.
**INTRODUCTION**			
Rationale	3	Describe the rationale for the review in the context of existing knowledge.	**Section 1, Introduction**<br>Describes clinical burden of ovarian endometriosis, limitations of conventional imaging, and rationale for radiomics.
Objectives	4	Provide an explicit statement of the objective(s) or question(s) the review addresses.	**Section 1, Introduction**<br>States objective to systematically review radiomics applications in ovarian endometriosis imaging.
**METHODS**			
Eligibility criteria	5	Specify the inclusion and exclusion criteria for the review and how studies were grouped for the syntheses.	**Section 2, Materials and Methods—eligibility-criteria paragraph**<br>Defines inclusion and exclusion criteria for study selection.
Information sources	6	Specify all databases, registers, websites, organisations, reference lists and other sources searched or consulted to identify studies. Specify the date when each source was last searched or consulted.	**Section 2, Materials and Methods—search-strategy paragraph**<br>Specifies PubMed, Scopus, and Web of Science databases and search timeframe.
Search strategy	7	Present the full search strategies for all databases, registers and websites, including any filters and limits used.	**Section 2, Materials and Methods—search-strategy paragraph**<br>see Supplementary File 1 for complete search strings.
Selection process	8	Specify the methods used to decide whether a study met the inclusion criteria of the review, including how many reviewers screened each record and each report retrieved, whether they worked independently, and if applicable, details of automation tools used in the process.	**Section 2, Materials and Methods—study-selection paragraph**<br>Describes independent screening by reviewers and consensus resolution process.
Data collection process	9	Specify the methods used to collect data from reports, including how many reviewers collected data from each report, whether they worked independently, any processes for obtaining or confirming data from study investigators, and if applicable, details of automation tools used in the process.	**Section 2, Materials and Methods—data-extraction paragraph**<br>Details independent data extraction and consensus verification.
Data items	10a	List and define all outcomes for which data were sought. Specify whether all results that were compatible with each	**Section 2, Materials and Methods—**

Section and Topic	Item #	Checklist item	Location where item is reported
		outcome domain in each study were sought (e.g. for all measures, time points, analyses), and if not, the methods used to decide which results to collect.	**data-extraction paragraph** Diagnostic performance metrics including accuracy, AUC, sensitivity, and specificity extracted.
	10b	List and define all other variables for which data were sought (e.g. participant and intervention characteristics, funding sources). Describe any assumptions made about any missing or unclear information.	**Section 2, Materials and Methods—data-extraction paragraph** Study characteristics, imaging modalities, and radiomics methodology extracted.
Study risk of bias assessment	11	Specify the methods used to assess risk of bias in the included studies, including details of the tool(s) used, how many reviewers assessed each study and whether they worked independently, and if applicable, details of automation tools used in the process.	**Section 2, Materials and Methods—risk-of-bias-assessment paragraph (QUADAS-2)** QUADAS-2 tool used to assess methodological quality. **See also Section 3.5** for a supplementary radiomics-specific methodological quality appraisal
Effect measures	12	Specify for each outcome the effect measure(s) (e.g. risk ratio, mean difference) used in the synthesis or presentation of results.	**Section 2, Materials and Methods—data-synthesis paragraph** Diagnostic accuracy, AUC, sensitivity, and specificity used as performance measures.
Synthesis methods	13a	Describe the processes used to decide which studies were eligible for each synthesis (e.g. tabulating the study intervention characteristics and comparing against the planned groups for each synthesis (item #5)).	**Section 2, Materials and Methods—data-synthesis paragraph** Qualitative synthesis approach described.
	13b	Describe any methods required to prepare the data for presentation or synthesis, such as handling of missing summary statistics, or data conversions.	**Section 2, Materials and Methods—data-synthesis paragraph** Extraction and comparison of radiomics performance metrics.
	13c	Describe any methods used to tabulate or visually display results of individual studies and syntheses.	**Section 3, Results → Tables 1–5 and Figures 1–5 (Sections 3.1–3.5)** Study characteristics, model performance, and risk of bias visualized.
	13d	Describe any methods used to synthesize results and provide a rationale for the choice(s). If meta-analysis was performed, describe the model(s), method(s) to identify the presence and extent of statistical heterogeneity, and software package(s) used.	**Section 2, Materials and Methods—data-synthesis paragraph** Qualitative comparison of radiomics models performed.
	13e	Describe any methods used to explore possible causes of heterogeneity among study results (e.g. subgroup analysis, meta-regression).	**Section 3.5, Radiomics performance synthesis,** Variability across imaging modalities and models described.

PRISMA 2020 Checklist

Section and Topic	Item #	Checklist item	Location where item is reported
	13f	Describe any sensitivity analyses conducted to assess robustness of the synthesized results.	**Not performed → qualitative synthesis only**
Reporting bias assessment	14	Describe any methods used to assess risk of bias due to missing results in a synthesis (arising from reporting biases).	**Section 5, Limitations**<br>No formal assessment of reporting bias was performed because of the small number and methodological heterogeneity of the included studies.
Certainty assessment	15	Describe any methods used to assess certainty (or confidence) in the body of evidence for an outcome.	**Section 5, Limitations**<br>No formal certainty-of-evidence framework was applied; the limitations and overall strength of the evidence are discussed qualitatively in Section 5.
**RESULTS**			
Study selection	16a	Describe the results of the search and selection process, from the number of records identified in the search to the number of studies included in the review, ideally using a flow diagram.	**Section 3.1, Study Selection and Characteristics (Figure 2)**<br>Number of screened, excluded, and included studies clearly reported.
	16b	Cite studies that might appear to meet the inclusion criteria, but which were excluded, and explain why they were excluded.	**Section 3.1, Study Selection and Characteristics**<br>Reasons for exclusion described.
Study characteristics	17	Cite each included study and present its characteristics.	**Section 3.2–3.4, Tables 2–4,**<br>Detailed study characteristics presented.
Risk of bias in studies	18	Present assessments of risk of bias for each included study.	**Section 3.1, QUADAS-2 analysis and Figures 3–4**<br>Risk of bias assessment presented.
Results of individual studies	19	For all outcomes, present, for each study: (a) summary statistics for each group (where appropriate) and (b) an effect estimate and its precision (e.g. confidence/credible interval), ideally using structured tables or plots.	**Sections 3.2–3.4, Radiomics model performance**<br>Individual study results and performance metrics presented.
Results of syntheses	20a	For each synthesis, briefly summarise the characteristics and risk of bias among contributing studies.	**Section 3.5, Radiomics performance synthesis**<br>Summary of diagnostic performance across studies.
	20b	Present results of all statistical syntheses conducted. If meta-analysis was done, present for each the summary estimate and its precision (e.g. confidence/credible interval) and measures of statistical heterogeneity. If comparing groups, describe the direction of the effect.	**Sections 3.2–3.5, Qualitative synthesis**<br>Comparative analysis of radiomics model performance.
	20c	Present results of all investigations of possible causes of heterogeneity among study results.	**Sections 3.2–3.4, Modality-specific performance comparison**<br>Differences across ultrasound, MRI, and CT radiomics discussed.

Section and Topic	Item #	Checklist item	Location where item is reported
	20d	Present results of all sensitivity analyses conducted to assess the robustness of the synthesized results.	**Not applicable;** No sensitivity analysis of the qualitative synthesis was performed.
Reporting biases	21	Present assessments of risk of bias due to missing results (arising from reporting biases) for each synthesis assessed.	**Section 5, Limitations** No formal assessment of reporting bias was performed because of the small number and methodological heterogeneity of the included studies.
Certainty of evidence	22	Present assessments of certainty (or confidence) in the body of evidence for each outcome assessed.	**Section 5, Limitations** No formal certainty-of-evidence framework was applied; the limitations and overall strength of the evidence are discussed qualitatively in Section 5.
**DISCUSSION**			
Discussion	23a	Provide a general interpretation of the results in the context of other evidence.	**Section 4, Discussion** Interpretation of radiomics performance and clinical implications.
	23b	Discuss any limitations of the evidence included in the review.	**Section 5, Limitations** Methodological and study limitations discussed.
	23c	Discuss any limitations of the review processes used.	**Section 5, Limitations** Limitations of systematic review process addressed.
	23d	Discuss implications of the results for practice, policy, and future research.	**Section 6, Conclusions** Clinical implications and future research directions described.
**OTHER INFORMATION**			
Registration and protocol	24a	Provide registration information for the review, including register name and registration number, or state that the review was not registered.	**Section 2, Materials and Methods—protocol and registration statement** Review protocol status described.
	24b	Indicate where the review protocol can be accessed, or state that a protocol was not prepared.	**Section 2, Materials and Methods—protocol and registration statement** Protocol information provided.
	24c	Describe and explain any amendments to information provided at registration or in the protocol.	**Section 2, Materials and Methods—protocol and registration statement** No protocol amendments reported.
Support	25	Describe sources of financial or non-financial support for the review, and the role of the funders or sponsors in the review.	**Funding statement** Financial support disclosed.
Competing interests	26	Declare any competing interests of review authors.	**Conflicts of Interest statement** Authors declare conflicts of interest status.

Section and Topic	Item #	Checklist item	Location where item is reported
Availability of data, code and other materials	27	Report which of the following are publicly available and where they can be found: template data collection forms; data extracted from included studies; data used for all analyses; analytic code; any other materials used in the review.	**Data Availability Statement** Availability of supporting data specified.

Table S2. Complete PRISMA 2020 checklist.

From: Page MJ, McKenzie JE, Bossuyt PM, Boutron I, Hoffmann TC, Mulrow CD, et al. The PRISMA 2020 statement: an updated guideline for reporting systematic reviews. BMJ 2021;372:n71. doi: 10.1136/bmj.n71. This work is licensed under CC BY 4.0. To view a copy of this license, visit https://creativecommons.org/licenses/by/4.0/
