# Supplementary material for: Radiomics and Artificial Intelligence in Ovarian Endometriosis Imaging: A Systematic Review and Critical Appraisal of Emerging Evidence
Source: Bioengineering (Basel). 2026 Jul 24;13(8):855. doi: 10.3390/bioengineering13080855 (PMC13509516; doi:10.3390/bioengineering13080855)
Supplement: Supplementary file 1 [file bioengineering-13-00855-s001.zip › Supplementary File 3.pdf]

	Risk of Bias					Applicability Concerns			
	Patient Selection	Index Test	Reference Standard	Flow and Timing	Key Notes	Patient Selection	Index Test	Reference Standard	Key Notes
Hu, 2023 [40]	Unclear	High	Low	Low	Retrospective pathology-based cohort; CNN development and optimization on internal data only; no external validation; histopathology as ground truth	Low	Unclear	Low	Clinically relevant ovarian endometriosis cohort; US-based deep learning with limited generalizability due to single-center design
Jiang, 2022 [46]	Unclear	High	Low	Unclear	Retrospective single-center cohort; AI algorithm evaluated on same dataset used for development; no external validation; histopathology as ground truth	Low	Unclear	Low	Clinically relevant ovarian endometriosis cohort; custom MRI-based AI workflow with limited generalizability
Li, 2023 [45]	Low	Unclear	Low	Low	Consecutive recruitment; single-center consecutive retrospective cohort; well-described CT radiomics workflow with internal validation; no external validation; histopathology as reference	Low	Low	Low	Clinically relevant ovarian cyst cohort; CT-based radiomics nomogram with single-center acquisition and manual segmentation
Li, 2024 [41]	Unclear	High	Low	Low	Retrospective single-center cohort; DL model optimized on internal dataset only; no external validation; expert-based reference standard	Low	Unclear	Unclear	Ovarian endometrioma population appropriate; US-based DL system with limited generalizability
Liu, 2024 [42]	Low	Unclear	Low	Low	Retrospective single-center study; consecutive cases; radiomics workflow well described; blinding not explicitly reported; partial follow-up–based diagnosis	Low	Low	Low	Representative population; manual segmentation; no external validation
Lupean, 2020 [25]	Unclear	Unclear	Unclear	Low	Retrospective pilot study; semi-automatic MRI texture analysis; partial blinding; mixed reference standard	Low	Unclear	Low	Clinically representative ovarian cyst population; single-center MRI protocol; texture analysis software limits generalizability
Podda, 2024 [43]	Unclear	High	Unclear	Low	Retrospective single-center cohort; DL segmentation model trained and evaluated on internal data only; expert annotation used as reference standard	Low	Unclear	Unclear	Pelvic/ovarian endometriosis population appropriate; US-based DL segmentation with limited generalizability and non-histopathological reference
Seo, 2021	Unclear	Low	Low	Low	Retrospective single-center	Unclear	Low	Low	Endometriotic cysts analyzed

[44]					cohort; CT texture analysis performed by blinded readers; histopathology as reference standard; appropriate imaging–surgery interval				within a broader benign ovarian cyst population; clinically realistic CT radiomics workflow
Ștefan, 2021 [39]	Unclear	Low	Low	Low	Retrospective single-center pilot study; ultrasound texture analysis with blinded segmentation; partial blinding during image selection; internal model development	Low	Low	Low	Representative ovarian cyst population; standard TVUS-based texture analysis; surgical histopathology as reference standard

Table S3. Detailed domain-level judgments and the rationale for each QUADAS-2 assessment.
