# Supplementary material for: Radiomics and Artificial Intelligence in Ovarian Endometriosis Imaging: A Systematic Review and Critical Appraisal of Emerging Evidence
Source: Bioengineering (Basel). 2026 Jul 24;13(8):855. doi: 10.3390/bioengineering13080855 (PMC13509516; doi:10.3390/bioengineering13080855)
Supplement: Supplementary file 1 [file bioengineering-13-00855-s001.zip › Supplementary File 4.pdf]

No.	Author, year	95% CI (AUC / Se / Sp / Acc)	External validation	Features before → after selection	Extraction software / IBSI	Calibration	Split level	DL leakage prevention
1	Ştefan et al., 2021 [39]	AUC 1.0 (0.918–1); Se 100% (88.4–100%); Sp 100% (75.3–100%)	None — no train/test split; whole cohort (n=56) used for both selection and evaluation	275 → 7 (final multivariate model)	MaZda (no IBSI); Topaz DeNoise AI (denoising)	Not reported	N/A	N/A (not DL)
2	Hu et al., 2023 [40]	AUC 0.986 (0.954–1); Acc 0.968 (0.838–0.998)	Yes — genuine temporal external cohort (171 train/31 test)	N/A — deep learning	N/A (deep features)	Figure labeled "calibration curve" (Fig. 3B) exists but never formally quantified in text	Patient-level	Explicitly mitigated — multi-image-per-patient averaging
3	Li et al., 2024 [41]	Not reported (mean ± SD across 5-fold CV)	Partial — 100-image hold-out from same overall pool, not independent	N/A — end-to-end deep learning	N/A	Not reported	1601 images/406 patients total; 1501 used for model development and reported evaluation, 100 held out	Not addressed in source paper
4	Liu et al., 2024 [42]	AUC 0.967 (0.940–0.995), test; nomogram 0.987 (0.967–1.000)	No independent external cohort; genuine patient-level split (326/81)	107 → 22 (LASSO)	PyRadiomics, IBSI-aligned	Yes — calibration curves + Hosmer-Lemeshow + DCA	Patient-level (1 lesion/patient)	N/A (ML, one image/patient)
5	Podda et al., 2024 [43]	Not reported (mean ± SD across 5-fold CV)	None — internal-only 5-fold CV, 80/20 split	N/A — pixel-wise DL segmentation	N/A	Not reported	Image-level (75 images; patient count not separately specified)	Not addressed in source paper

Table S4: Extended per-study methodological detail for the ultrasound-based radiomics studies included in this review, supplementing Table 2.

AUC = area under the curve; Se = sensitivity; Sp = specificity; Acc = accuracy; CI = confidence interval; DL = deep learning; IBSI = Image Biomarker Standardisation Initiative; ICC = intraclass correlation coefficient; LASSO = least absolute shrinkage and selection operator.

No.	Author, year	95% CI	External validation	Features before → after selection	Extraction software / IBSI	Calibration	Split level	DL leakage prevention
1	Seo et al., 2021 [44]	Not reported anywhere — no AUC/ROC analysis performed	Not genuine — decision trees built on Reviewer 1's data; Reviewer 2's readings applied to the same trees on the same 135 patients	42 → 2 (final decision trees)	TexRAD, no IBSI compliance	None	No formal split — same 135 patients throughout	N/A (not DL)
2	Li et al., 2023 [45]	Nomogram: 0.925 (0.885–0.965) train, 0.942 (0.891–0.993) validation; clinical 0.866/0.831; radiomics signature 0.841/0.802	No independent external cohort; genuine patient-level split (200/87)	1316 → 1109 (ICC>0.8) → 289 (Wilcoxon $p<0.05$) → 17 (LASSO)	PyRadiomics, IBSI-aligned ($1\times1\times1$ mm^3, 25 HU bins)	Yes — calibration curves (Supp. Fig. S4a–f), follow the 45° line	Patient-level (200/87)	N/A

Table S5: Extended per-study methodological detail for the computed tomography-based radiomics studies included in this review, supplementing Table 3.

AUC = area under the curve; Se = sensitivity; Sp = specificity; Acc = accuracy; CI = confidence interval; DL = deep learning; IBSI = Image Biomarker Standardisation Initiative; ICC = intraclass correlation coefficient; LASSO = least absolute shrinkage and selection operator.

No.	Author, year	95% CI	External validation	Features before → after selection	Extraction software / IBSI	Calibration	Split level	DL leakage prevention
1	Lupean et al., 2020 [25]	AUC 1.0 (0.9–1); Se 100% (85.8–100%); Sp 100% (71.5–100%)	None — explicitly no split (n=43 for both)	>300 (342 per Table 4) → 14 (final model; 1 confirmed independent multivariate predictor)	MaZda	Not reported	N/A	N/A
2	Jiang et al., 2022 [46]	Not reported (mean ± SD)	None identified — the 116-patient control/experimental split is a nursing-intervention comparison arm, unrelated to FCM/HCM algorithm validation	N/A — Conventional clustering-based image segmentation without radiomic feature extraction	N/A	None	No clear split identified for the segmentation comparison	N/A (unsupervised clustering)

Table S6: Extended per-study methodological detail for the magnetic resonance imaging-based radiomics studies included in this review, supplementing Table 4.

AUC = area under the curve; Se = sensitivity; Sp = specificity; Acc = accuracy; CI = confidence interval; DL = deep learning; IBSI = Image Biomarker Standardisation Initiative; ICC = intraclass correlation coefficient; LASSO = least absolute shrinkage and selection operator.
